# Supplementary material for: Trophic position of Otodus megalodon and great white sharks through time revealed by zinc isotopes
Source: Nat Commun. 2022 May 31;13:2980. doi: 10.1038/s41467-022-30528-9 (PMC9156768; doi:10.1038/s41467-022-30528-9)
Supplement: Supplementary file 1 — Supplementary Information [file 41467_2022_30528_MOESM1_ESM.pdf]

Supplementary Information for:

Trophic position of *Otodus megalodon* and great white sharks through time revealed by zinc isotopes

Jeremy McCormack, Michael L. Griffiths, Sora L. Kim, Kenshu Shimada, Molly Karnes, Harry Maisch IV, Sarah Pederzani, Nicolas Bourgon, Klervia Jaouen, Martin A. Becker, Niels Jöns, Guy Sisma-Ventura, Nicolas Straube, Jürgen Pollerspöck, Jean-Jacques Hublin, Robert A. Eagle and Thomas Tütken

This PDF file contains:

Supplementary Notes

Supplementary Note 1

Supplementary Note 2

Supplementary Methods

Supplementary Discussion

Supplementary Discussion 1

Supplementary Discussion 2

Supplementary Figures 1-12

Supplementary Tables 1-12

Supplementary References

Supplementary Notes

Supplementary Note 1

In most cases, stable isotope analysis of fossil remains is the best option for studying the overall diet and ecology of extinct animals[1]. Nitrogen (N) isotope analysis ($\delta^{15}N$) of fossil bone or dentine collagen is a well-established method indicating a species' trophic position, but this method is limited by the degree of protein preservation and is rarely applicable to fossils older than ~100,000 years[2]. Only during the past 10 to 15 years, non-traditional isotope systems such as calcium and zinc (Zn) in bones and teeth were recognised as potential/new palaeodietary and trophic level indicators in the terrestrial[3-5] and marine realms[6-8].

Most ecological studies report the Zn isotope ratio of the two most abundant isotopes $^{66}Zn/^{64}Zn$ expressed as $\delta^{66}Zn$ value relative to the JMC-Lyon standard solution[4,5,7,8]. Zinc isotopes can be used as ecological proxies because Zn is highly fractionated within organisms, controlled by the coordination of Zn with different ligands. Heavy Zn isotopes are concentrated in stiffer bonds involving oxygen in hydroxides, phosphates and carbonates and nitrogen in histidine, whereas sulphides are enriched in lighter Zn[9]. For example, heavy Zn is typically enriched in mammal bones because of Zn binding to phosphates, whereas the liver is enriched in light Zn isotopes due to its bond to sulphur in cysteine-rich proteins[9,10]. As a result, muscles and most organs are typically ^{66}Zn depleted relative to the animal's diet[9-11] resulting in successively lower $\delta^{66}Zn$ value with increasing trophic level. Per trophic level, bioapatite $\delta^{66}Zn$ values decrease presumably by approximately 0.4 ‰ in marine and terrestrial mammals[4,8]. However, the exact trophic level fractionation factors have yet to be experimentally determined and may or may not vary depending on the consumer and/or bioavailability of Zn in the food item.

Most non-diet related factors, such as sex and age of an animal, have shown no noticeable effect on the Zn isotope values and relative isotopic variability in body tissues[11,12]. Not only does $\delta^{66}Zn$ offer the potential to act as an independent additional trophic level indicator to verify traditional $\delta^{15}N$ results in ecological studies, but it also offers additional advantages. Recent studies have suggested that $\delta^{66}Zn$ can identify specific feeding behaviours such as the consumption of bones or omnivory[4,5,13]. For marine mammals, $\delta^{66}Zn$ values within a species are much more homogenous geographically than $\delta^{15}N$ and $\delta^{13}C$ (at least within the Arctic), indicating a potentially lower isotopic variability at the base of the food web[8]. Consequently, zinc isotope analysis may allow for a more direct comparability of marine species trophic ecology between spatially and temporally distinct locations than possible by traditional isotope analysis alone.

The isotopic composition of marine dissolved Zn below 500 m seems to be globally homogenous with values close to +0.5 ‰, despite variable Zn concentrations[14,15]. Consequently, the bulk isotopic composition of dissolved marine Zn is enriched in ^{66}Zn relative to its major inputs from rivers and aeolian dust, which centre on the global crustal average of +0.3 ‰[16,17]. Most oceans show a nutrient-like vertical distribution of dissolved Zn concentration due to biological uptake, leading to highly depleted surface waters with concentrations often less than 1 nmol kg^{-1} (e.g., ref.[18-20]). Contrary to the deep water, particularly in the uppermost surface water (< 20 m depth), dissolved Zn isotope values vary significantly, whereby values between −1.1 to +0.9 ‰ and −0.9 and +0.2 ‰ can be found across a North Atlantic and North Pacific transect, respectively[19,21]. This heterogeneity in dissolved Zn isotope values contrasts with the relatively homogenous $\delta^{66}Zn$ values observed within marine mammals across the Arctic[8]. Importantly, however, there is hardly any data on the Zn isotope composition of natural marine planktonic organisms[22,23]. Therefore, the $\delta^{66}Zn$ composition of marine food web baselines, i.e., Zn isotope values of particulate organic matter, remains poorly understood.

Supplementary Note 2

Details on the extant and fossil material used in this study (including museum catalogue numbers, locality, age, and stratigraphic context) can be found in Supplementary Data 1.

Baden-Württemberg, Germany

All fossils from Baden-Württemberg are from the sandpits Walbertsweiler and Rengetsweiler, which are both located between the cities Messkirch and Pfullendorf (Sigmaringen County).

Within the Ottnangian the marine transgression of the western Paratethys started from a southwestern direction. The transgression manifests in approximately 50 m thick glauconitic sands of the Heidenlöcher Schichten[24]. The 20 to 70 m thick Kalkofen Formation is overlying these layers. The Kalkofen Formation consists in its lower part of glauconitic sands and marls; the upper part is formed by a sequence of marls showing only minor glauconitic sand fractions (lenses)[25]. The Baltringen Formation is overlying the Kalkofen Formation[26]. Both the type locality (abandoned stone pit Baltringen[27]) as well as other outcrops of the Baltringen Formation in southern Germany are composed of a conglomeratic sandstone with a maximum thickness of 2.5 m with a coarsely to medium granular texture followed by fine to medium granular sands, called the 'Baltringer Horizon'. The Baltringer Horizon was deposited in a shallow to coastal marine area[25,26,28]. Despite granite debris, the Baltringer Horizon contains multiple macrofossils, for example, invertebrate fossils, such as clamshells, and teeth of terrestrial and marine vertebrates including sharks and rays[29-32].

Sandpit Walbertsweiler:

Samples analysed herein were collected from both the Kalkofen Formation as well as the Baltringer Formation.

Today, Walbertsweiler is a recultivated sandpit, where a 1.6 m thick segment of the Kalkofen Formation is accessible. The particular profile of the site shows an interchanging sequence of little compacted marl and coarsely granular sands[29]. Organic debris and bedding planes with ripple marks point to a coastal, inner neritic to shallow marine environment. The water depth is estimated to have been less than 50 m based on the frequency of shallow water foraminifers[25,28]. The former coastline was located north, approximately less than 25 km away. The profile was situated 10 m below the Baltringer Horizon (Baltringen Fm.). The Baltringer Horizon was formerly also accessible at this site.

Sediments of the Kalkhofen Formation are assigned to the Lower Ottnangian (Middle Burdigalian). All overlaying deposits are assigned to the Baltringer Formation and formed in the Middle Ottnangian (Middle Burdigalian).

Sandpit Rengetsweiler:

Samples analysed herein were collected from the Baltringer Formation. Sediment attached to teeth was also sampled from Rengetsweiler (n = 2).

The Rengetsweiler sandpit is neighbouring the Walbertsweiler sandpit and is still in use today. Here, only the Baltringer Formation sediments are accessible. See the geological background of the Walbertsweiler sandpit for details.

Gozo, Republic of Malta

Malta and Gozo are part of the carbonate platform, stretching from south-eastern Sicily (Italy) to Tunisia and West-Lybia. This carbonate platform represents the foreland of the Apennine-Sicilian-Maghrebian belt[33]. The accessible sediments span from the Oligocene to the upper Miocene. In detail, the different formations are[34-36]:

a. Lower Coralline Limestone Formation (Oligocene, Chattian)
b. Globigerina limestone:
   - Lower Globigerina limestone (Late Oligocene, Upper Chattian)
   - phosphorite bed C1 (Lower Burdigalian)
   - Middle Globigerina limestones (Burdigalian)
   - phosphorite bed C2 (Upper Burdigalian)
   - Upper Globigerina limestone (Langhian)
c. Blue Clay Formation (Serravallian)
d. Greensand (Tortonian)
e. Upper Coralline Limestone, Tortonian

The sampled material was collected in 1988 exclusively from the surface of the phosphorite layer C2 (b). Details on the selachian and batoid fauna can be found in Menesini[37] and Ward & Bonavia[38].

North Carolina (NC), USA, submerged continental shelf, Onslow Bay and land-based exposures at "Lee Creek"

Onslow Bay:

The Cenozoic marine sediments of Onslow Bay cover a high-relief crystalline basement, the Carolina Platform and the mid-Carolina Platform High, also known as the Cape Fear Arch[39-42]. Across much of Onslow Bay, sediments of the Miocene Pungo River Formation and the Pliocene Yorktown Formation occur at or close to the seafloor[43-46].

Fossil teeth were collected from the submerged continental shelf of Onslow Bay while SCUBA diving. These teeth are from localities previously described in Maisch et al.[45,46] and belong to the Pungo River Formation (Burdigalian) and the Yorktown Formation (Early Pliocene, Zanclean). The samples were collected on the gently sloping continental shelf of southwestern Onslow Bay approximately 30 km, 40 km, and 60 km from the present shoreline, at depths of approximately 25 m, 30 m, and 35 m, respectively[45]. All submerged shelf localities consist of low-relief hardbottom scarps (<1000 m^2) with adjacent lag deposits composed of well-rounded and poorly sorted cobbles, pebbles, and sand-sized clasts that contain an abundance of megatoothed shark teeth. Limestone scarp and underlying brown-grey clay at the shallower shelf locality belong to the Pungo River Formation, whereas the limestone scarp with underlying grey-blue clay present at the intermediate and deeper shelf localities can be assigned to the Yorktown Formation[45-49].

"Lee Creek", Nutrien Phosphate Mine, Aurora, NC:

Taxa in the Onslow Bay assemblage are also known from land-based exposures along the Atlantic Coastal Plain of the United States[48,50-52]. The Nutrien Phosphate Mine (i.e., Lee Creek; PCS Mine; Aurora Mine) near Aurora, NC, northeast of Onslow Bay, contains land-based exposures of the Miocene Pungo River Formation and Pliocene Yorktown Formations as well as a nearly identical vertebrate fossil assemblage to that occurring in Onslow Bay, NC[45,48]. Samples analysed herein from land-based exposures at the Nutrien Phosphate Mine belong to the Burdigalian Pungo River Formation.

Florida, USA, submerged continental shelf off Venice and land-based exposures in the Peace River, near Wauchula

Venice:

The shallow continental shelf off the coast of Venice, Florida, contains Cenozoic marine sediments that become progressively younger to the south[53,54]. These sediments consist of the Bone Valley Member of the Peace River Formation (Late Miocene-Early Pliocene) and the Tamiami Formation (Pliocene)[53]. Extensive deposition and erosion of these sediments has occurred over the last approximately 12 million years in response to numerous transgressive–regressive glacioeustatic sea-level cyclicity

events, wave-based erosion and ocean currents forming fossiliferous lag deposits on the modern seafloor[55,56].

The lag deposits consist of low-relief hardbottom scarps and adjacent rubble zones of limestone and vertebrate fossil clasts or hummocky black, phosphatic sand rows in ≤ 12 m depth, within 3 km of the modern-day shoreline. The Miocene-Pliocene Bone Valley Member of the Peace River Formation exposed off the coast of Venice consists of grey clays containing an abundance of phosphorite. In contrast, the Tamiami Formation exposed off the coast of Venice consists of white-light grey coloured dolomite and limestone hardbottom scarps with a lesser amount of sand-sized phosphorite grains. These identifications are also consistent with the stratigraphic and formation boundaries of substrate sediments along the west coast of central Florida constructed by Scott[57,58] and Scott et al.[53].

Peace River, near Wauchula:

Similar to the submerged Miocene-Pliocene deposits off the coast of Venice, FL, those exposed inland along the Peace River and in other locations across central Florida are nearly identical[53,56,59]. In these locations, the Peace River Formation is generally a white-yellow coloured sandy limestone with abundant, sand-sized phosphorite grains, which locally exposes grey-blue coloured clays, while the Tamiami Formation is a shelly, white-grey dolomite-calcitic limey sand[54,56,60-62].

Nagasaki-hana, Choshi City, Chiba Prefecture, Japan

The Japanese samples of *Otodus megalodon* examined in this study come from the Early Pliocene Na-Arai Formation at Nagasaki-hana in Choshi City, Chiba Prefecture. The occurrences of teeth of *O. megalodon* from the formation were reported by Itoigawa et al.[63]. The Na-Arai Formation is represented by a marine conglomerate deposit, and microfossils suggest a minimum age of 4.36 Ma[64]. Boessenecker et al.[65] considered the age of the Na-Arai Formation to be 5.33-4.36 Ma.

Supplementary Methods

Enameloid powders were converted to silver phosphate (Ag_3PO_4) for oxygen isotope measurements of bioapatite phosphate following an adapted version of the rapid precipitation protocol developed by Dettman et al.[66] and modified by Tütken et al.[67]. Approximately 5 mg of each enameloid sample were weighed into 2 mL Eppendorf microcentrifuge tubes. Samples were agitated in 0.4 mL of 2 M hydrofluoric acid (HF) for 24 hours to digest the bioapatite and remove calcium from the solution as calcium fluoride precipitate (CaF_2). The phosphate-containing solution and the CaF_2 precipitate were separated by centrifugation (12000 rpm for 5 min) and the sample solution was transferred to a clean microcentrifuge tube. In order to maximise phosphate recovery, the CaF_2 precipitate was washed once with 0.1 mL MilliQ ultrapure water, and the wash was added to the phosphate-containing solution. The sample solution was then titrated to neutrality as indicated by the colour change point of Bromthymolblue indicator using 25 % ammonia solution (NH_4OH). From the neutralised solutions, Ag_3PO_4 was crash precipitated by the addition of 0.4 mL 2 M silver nitrate ($AgNO_3$) solution. The resulting precipitate was pelleted by centrifugation (12000 rpm for 5 min), and the remaining silver nitrate solution was removed. The silver phosphate was then washed three times with MilliQ ultrapure water using centrifugation and vortex mixing steps between rinses to eliminate any remaining silver nitrate from the sample. Silver phosphate samples were then dried overnight at 50 °C and stored over desiccant until further analysis. Phosphate recovery of estimated original phosphate content of enameloid was 87 % on average, assuming a phosphate content of 51.55 % in shark enameloid, as published for *Galeocerdo cuvier*[68]. Given the variability in shark enameloid phosphate content and the lack of relationship between phosphate yield and $\delta^{18}O$ values, these yields are consistent with a quantitative conversion to Ag_3PO_4.

Oxygen isotope delta measurements of Ag_3PO_4 were conducted using a high-temperature elemental analyser (TC/EA) coupled to a Delta V isotope ratio mass spectrometer via a Conflo IV interface

(Thermo Fisher Scientific, Bremen, Germany) at the Max-Planck-Institute for Evolutionary Anthropology (MPI-EVA). Approximately 0.5 mg of each silver phosphate sample was weighed into cleaned silver capsules (3x4 mm, IVA Analysentechnik, Meerbusch, Germany) and introduced to the TC/EA using a Costech Zero Blank Autosampler (Costech International, Cernusco sul Naviglio, Italy). High-temperature conversion to CO was achieved using a reactor temperature of 1450 °C, and gases were separated using a Eurovector E11521 1.4 m x 4 mm x 6 mm stainless steel GC column with 80/100 mesh 5 Å molecular sieve packing (Eurovector Instruments & Software, Pavia, Italy) maintained at 120 °C with a carrier gas pressure of 1.3 bar. Some of the extant enameloid samples showed a detectable N_2 peak, but a separation of more than 90 seconds between the N_2 and CO peak was achieved, and $\delta^{18}O$ values of these individuals were consistent with other individuals from the same species.

Oxygen isotope delta values were two-point scale normalised to the VSMOW scale using matrix-matched standards calibrated to international reference materials. Scale normalisation was checked using three different quality control standards. Scale normalisation was conducted using the B2207 silver phosphate standard ($\delta^{18}O$ = 21.7 ± 0.3 ‰, 1 SD; Elemental Microanalysis, Okehampton, UK) and an in-house silver phosphate standard (KDHP.N, $\delta^{18}O$ = 4.2 ± 0.3 ‰, 1 SD). This in-house standard was obtained by equilibrating a KH_2PO_4 solution with Leipzig winter precipitation at ca. 140 °C for several days, after which the solution was neutralised using a small amount of NH_4OH, and the phosphate precipitated as silver phosphate by addition of $AgNO_3$ solution. The accepted value of this in-house standard was determined by two-point calibration using B2207 and IAEA-SO-6 (barium sulphate, $\delta^{18}O$ = -11.35 ± 0.3 ‰, 1 SD)[69].

Samples were measured in triplicate, but in rare cases, individual measurements were rejected if they did not meet quality control criteria such as appropriate peak area to sample amount relationship. In such cases, $\delta^{18}O$ are therefore only based on two replicates. The average reproducibility of sample replicate measurements was 0.3 ‰. Consecutive analysis of sets of standards with widely spaced isotopic values showed no detectable memory effect, and consequently, no memory effect correction was used. No effect of the blank, sample amount, or peak height on the results was observed, and consequently, no blank correction or linearity correction was used. Repeated analysis of the quality control standard AS337382 in each run did not show any substantial within-run drift, and therefore no drift correction was applied.

Supplementary Discussion

Supplementary Discussion 1

There is generally a correlation between trophic level, as reported on FishBase[70], and bioapatite $\delta^{66}Zn$ values, which is also observed for the classic trophic level indicator $\delta^{15}N_{coll}$ (Supplementary Figure 1). The lowest $\delta^{66}Zn$ values are found in large predatory epipelagic to mesopelagic sharks (e.g., *Carcharodon carcharias*, *Isurus oxyrinchus*, *Galeocerdo cuvier*, *Carcharias taurus*, *Lamna ditropis*). The highest $\delta^{66}Zn$ values in enameloid were measured in lower trophic level teleosts (Figure 1). The highest $\delta^{66}Zn$ values from elasmobranchs were recorded in the basking shark (*Cetorhinus maximus*) measured not in enameloid but bulk gill rakers. As such, these values may not be directly comparable to enameloid. The gill rakers are composed of both pallial dentine and a thin enameloid cover[71]. We are unaware of any study investigating Zn concentrations among the tissue types of a gill raker. However, as dentine has a predictable mean offset of +0.2 ‰ relative to enameloid (Supplementary Figure 11), bulk gill raker should have values likely less than 0.2 ‰ higher compared to enameloid formed under the same conditions, which is still a higher $\delta^{66}Zn$ value than observed in enameloid of other elasmobranchs.

In comparison to the more offshore epipelagic apex predators (e.g., *C. carcharias*, *I. oxyrinchus*, *L. ditropis, G. cuvier*), the $\delta^{66}Zn$ values of *Carcharhinus* species are higher than expected (Figure 2). For

example, stomach content and soft tissue $\delta^{15}N$ values typically place *Carcharhinus leucas* on a similar trophic level as *G. cuvier*[72]. Similarly, KZN dentine $\delta^{15}N_{coll}$ values herein also demonstrate comparable values between *G. cuvier* and *Carcharhinus* species, with the former even having lower mean values (13.3 ‰) in comparison to the latter (14.2 ‰; Supplementary Figure 4).

The *Carcharhinus* species studied here all inhabit neritic waters[73] and feed primarily on demersal/benthic, freshwater-brackish-coastal prey[74-77]. Inshore/benthic food webs might differ in their baseline $\delta^{66}Zn$ value compared to pelagic plankton-based food webs, as observed for $\delta^{15}N$ and $\delta^{13}C$[78,79]. In particular, coastal food webs can be composed of multiple food chains, which, to varying degrees, rely on different nutrition sources, including autochthonous, local primary production, and allochthonous, transported from adjacent habitats[80]. For the KZN Bight, demersal organisms depend primarily on terrestrial riverine organic matter (OM) input, whereas offshore pelagic communities are supported by marine OM[81,82]. Differences in tissue isotopic compositions based on habitat-dependent diets were observed for muscle and blood $\delta^{13}C$ and mercury isotope values of sympatric bull sharks (*Carcharhinus leucas*) and *G. cuvier*, with the former species feeding on coastal demersal and the later mesopelagic prey[72,83]. However, $\delta^{13}C_{coll}$ values herein do not indicate more terrestrial $\delta^{13}C$ values in *Carcharhinus* species compared to *G. cuvier* and/or *Carcharodon carcharias* (Supplementary Figure 3). It is worth noting that while differences between a neritic and oceanic-based diet might skew $\delta^{66}Zn$-based trophic level determinations, differences between neritic and oceanic $\delta^{15}N$ baselines are well documented, e.g., with higher $\delta^{15}N$ values for neritic compared to oceanic zooplankton in the Gulf of Mexico[79].

Another possible explanation for the $\delta^{66}Zn$ differences observed between the KZN shark species could be taxon-dependent diet-bioapatite Zn discrimination factors. However, enameloid $\delta^{66}Zn$ values from sandbar sharks (*Carcharhinus plumbeus*) from aquariums are lower than wild *Carcharhinus*. They even overlap with the values of captive *C. taurus* (Figure 1, 3), implying that influences other than taxonomic discrimination factors at least contribute to the higher wild *Carcharhinus* values. We observe a strong internal biological control on Zn concentration ([Zn]) that is importantly independent of the $\delta^{66}Zn$ values. Enameloid [Zn] is variable between different taxonomic groups with carcharhiniformes generally having higher [Zn] than lamniformes (Supplementary Figure 5). Still, not all carcharhiniformes have systematically higher $\delta^{66}Zn$ values (e.g., *G. cuvier*). However, [Zn] in shark teeth can also vary within the enameloid of a single tooth without variation in $\delta^{66}Zn$, as observed here for a single *C. carcharias* tooth, with the highest [Zn] at the apex and lowest close to the root (respectively, 691 and 168 µg/g; Supplementary Figure 10). Additionally, $\delta^{66}Zn$ in dentine generally shows a similar species-specific variability as observed in enameloid with a relatively constant positive offset of $\Delta^{66}Zn_{d\text{-}e} \approx +0.22 \pm 0.1$ ‰ (n = 23) relative to enameloid of the same tooth (Supplementary Figure 11). Zinc concentrations ([Zn]) are typically higher in enameloid than in dentine (51 ± 20 µg/g, n = 23) by a factor of 10 to 10^2, but we observe no correlation between [Zn] and $\delta^{66}Zn$ values in neither tissue (Supplementary Figure 5d).

Noteworthy, due to the sampling of 5 to 10 mg of enameloid for $\delta^{66}Zn$ analyses, smaller lamniform (e.g., *Carcharias*, *Mitsukurina*, *Pseudocarcharias*) and carcharhiniform (e.g., *Galeocerdo*, *Hemipristis*, *Carcharhinus*) extant and fossil teeth are more likely to represent mean [Zn] along the tooth axis. In contrast, large lamniform (*Otodus*, *Carcharodon*) shark [Zn] presented here may be more skewed towards higher [Zn] due to preferential sampling of the apex area (Supplementary Figure 5).

While enameloid Zn concentration appears largely dependent on the taxonomic group and relative sampling position along the tooth crown, the same cannot be said for $\delta^{66}Zn$ values, arguing for the independence of [Zn] and $\delta^{66}Zn$ in both enameloid and dentine. Possible differences in $\delta^{66}Zn$ discrimination factors between shark species are likely unrelated to enzymatic processes during apatite crystal growth or differences in enameloid structure, which likely control [Zn] in elasmobranch and mammal enameloid[84,85]. Therefore, if species-specific isotopic discrimination occurs, it is more likely to be related to processes during gastrointestinal Zn absorption. However, the site and mode of gastrointestinal Zn absorption appear to be similar between fish and mammals[86], and there is no

indication of species-specific isotopic diet-bioapatite fractionation of Zn among mammal species. Still, taxonomically different discrimination factors for $\delta^{66}Zn$ cannot be entirely excluded here and require assessment in future studies.

Supplementary Discussion 2

The first and most striking argument against significant diagenetic alteration of pristine dietary $\delta^{66}Zn$ values for all fossil sites is the remarkable similarity in enameloid $\delta^{66}Zn$ values and [Zn] between fossil sites and with extant shark taxa (Figure 3, Supplementary Figure 5). The genus *Galeocerdo* covers the same range of $\delta^{66}Zn$ values and Zn concentrations among sites and geologic ages. Additionally, in all Miocene assemblages, the apex predator megatooth-shark *O. chubutensis* has mean $\delta^{66}Zn$ values amongst the lowest within each assemblage. *Carcharhinus* enameloid $\delta^{66}Zn$ values from Gozo and Miocene and Pliocene North Carolina are higher than in respective sympatric sharks, as observed for extant *Carcharhinus* taxa (Figure 3).

Identifying a possible exchange of fossil enameloid Zn with the diagenetic environment is challenging but may also be possible by comparing $\delta^{66}Zn$ values between enameloid and dentine of the same teeth. In extant fish teeth, dentine $\delta^{66}Zn$ values are on average 0.22 ‰ more positive than in enameloid ($\Delta^{66}Zn_{d\text{-}e} \approx +0.22 \pm 0.10$ ‰, n = 23), similar to the 0.2 ‰ higher values observed in bone relative to the enamel of terrestrial mammals[4]. Therefore, this enrichment in ^{66}Zn in dentine/bone relative to enameloid tissue is most likely due to differences in fractionation factors among the tissue types, which importantly appears to be taxon and [Zn] independent (Supplementary Figure 5, 11).

The higher porosity and smaller crystal size of dentine relative to enameloid make this tissue type more susceptible to diagenetic trace element exchange[87,88]. Additionally, the Zn concentration in most species analysed here tends to be enriched in modern and fossil enameloid relative to dentine by the factor 10 to 10^2 (Supplementary Figure 5d). In contrast, Zn concentrations in modern osteodentine (51 ± 20 µg/g, n = 23) are similar to those of the embedding carbonate sediment (≈ 23 to 58 µg/g), making the dentine $\delta^{66}Zn$ composition significantly more vulnerable to post mortem alteration. In fossil shark teeth, $\Delta^{66}Zn_{d\text{-}e}$ exceeds +0.2 ‰, with values between +0.42 to +1.66 ‰ (mean of +0.78 ± 0.33 ‰, n = 13). While these higher fossil $\Delta^{66}Zn_{d\text{-}e}$ values do not necessarily indicate pristine enameloid $\delta^{66}Zn$ values, they indicate that dentine $\delta^{66}Zn$ shifts to more positive values and, by implication, that enameloid is less diagenetically altered. Some dentine $\delta^{66}Zn$ values are even more positive than their embedding carbonate sediment (+0.34 to +0.49 ‰, Supplementary Figure 6), which lies close to the mean continental crustal value of +0.3 ‰[16].

Mean osteodentine [Zn] is also slightly lower in fossil (41 ± 13 µg/g, n = 13) than in extant teeth. Rather than Zn uptake, diagenetic alteration of bioapatite $\delta^{66}Zn$ values in these sites is likely due to Zn^{2+} replacement by Ca^{2+} (and/or other cations) with a preferential replacement of the lighter Zn isotopes. This preferential replacement is in line with general equilibrium isotope fractionation considerations dictating a higher amount of energy required to break the stiffer bond of the heavier isotope (^{66}Zn) compared to the lighter one (^{64}Zn)[89]. Diagenetically modified $\delta^{66}Zn$ values in dentine most likely result from Zn exchange within the bioapatite crystal lattice (and diagenetic environment), solid-state Zn diffusion and/or bioapatite dissolution and reprecipitation, explaining the higher $\delta^{66}Zn$ values in some dentine samples than in the surrounding sediment. These processes are more likely to affect dentine than enameloid, due to the larger surface-to-volume ratio of the bioapatite crystals and the higher tissue porosity[2].

Concurrent with this hypothesis, the sampling of enameloid along fractures compared to sampling along more 'pristine appearing' enameloid of the same tooth shows that the former is characterised by lower [Zn] and higher $\delta^{66}Zn$ values than the latter (Supplementary Figure 9). However, diagenetic zinc exchange cannot be estimated based on [Zn] alone, as [Zn] can vary significantly among teeth of the same species and within a single tooth, from apex to base (Supplementary Figure 10). Nonetheless,

as increased diagenetic Zn exchange leads to lower [Zn] and higher $\delta^{66}Zn$ values, the low [Zn] and higher $\delta^{66}Zn$ values in the surrounding sediment and the high [Zn] and low $\delta^{66}Zn$ values in fossil enameloid suggests that most samples experienced very little to no alteration of the original $\delta^{66}Zn$ values.

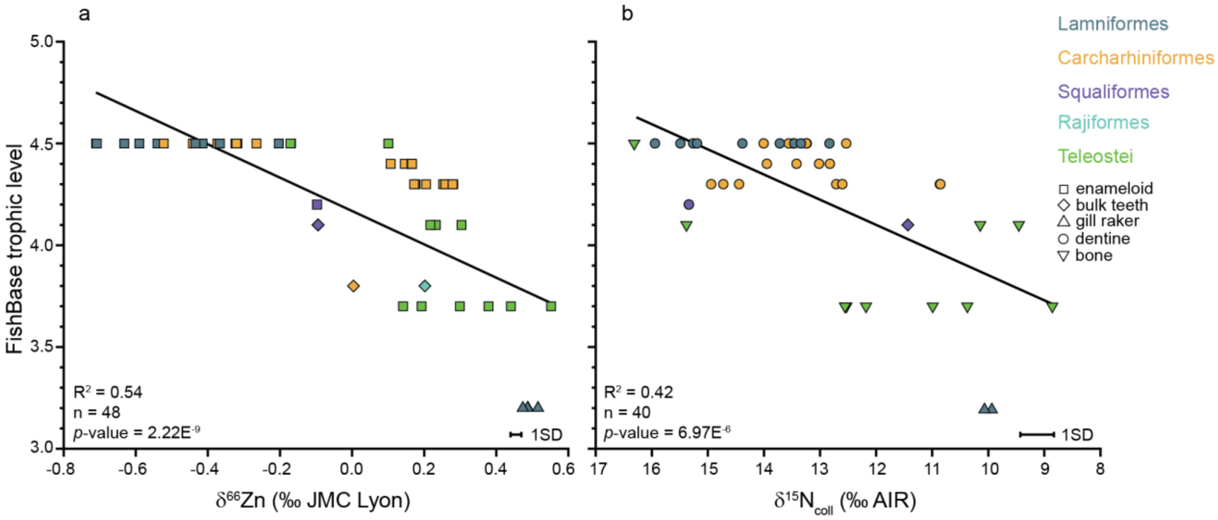

Supplementary Figure 1: Correlation between zinc (a) and nitrogen (b) isotope values of extant elasmobranch and teleost teeth and gill raker versus mean species trophic level as indicated on FishBase[70] based on diet studies. Controlled fed individuals from aquariums and the pisciculture were omitted. Animals are grouped and colour-coded following their taxonomic order. Note a correlation between a) $\delta^{66}Zn$ and trophic level and b) $\delta^{15}N_{coll}$ and trophic level despite the differences in geographic locations for the individual species (linear regression lines are shown). Note that $\delta^{15}N_{coll}$ is shown on an inverted x-axis for easier comparison to $\delta^{66}Zn$ (a). Note that depicted *r* and *p*-values were determined by Spearman correlation. Measurement uncertainty is indicated at the 1 SD level. Source data are provided as a Source Data file.

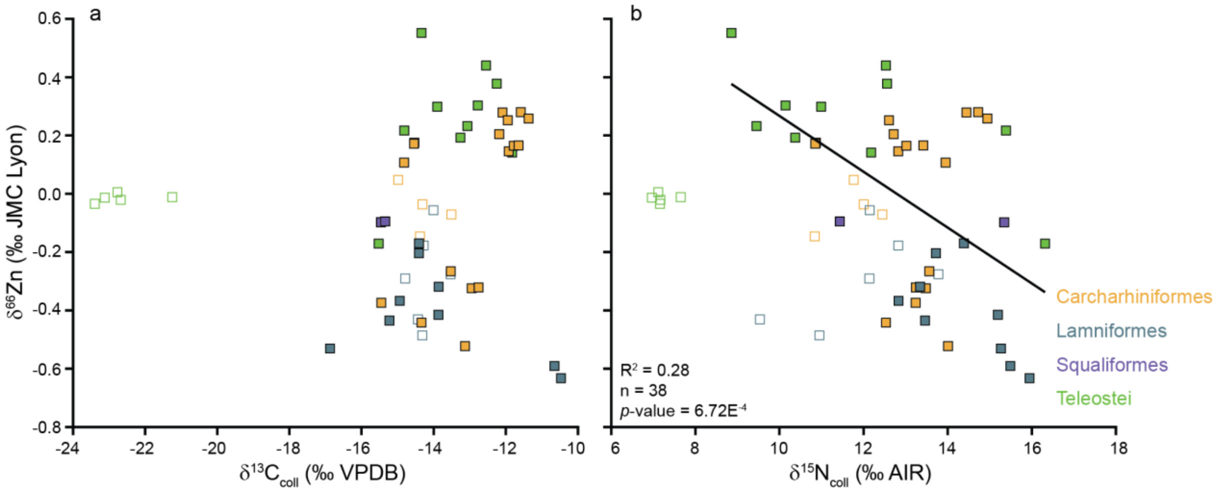

Supplementary Figure 2: Relationship between zinc isotope values and organic carbon (a) and nitrogen (b) isotope values of extant elasmobranch and teleost fish teeth and bone. Animals are colour-coded following their taxonomic order. Artificially fed aquarium sharks and pisciculture teleosts are indicated by open symbols. Note that the weak correlation between $\delta^{66}Zn$ and $\delta^{15}N_{coll}$ is for wild species only (i.e., aquarium sharks and pisciculture teleosts are excluded). The linear regression line is shown in (b). Source data are provided as a Source Data file.

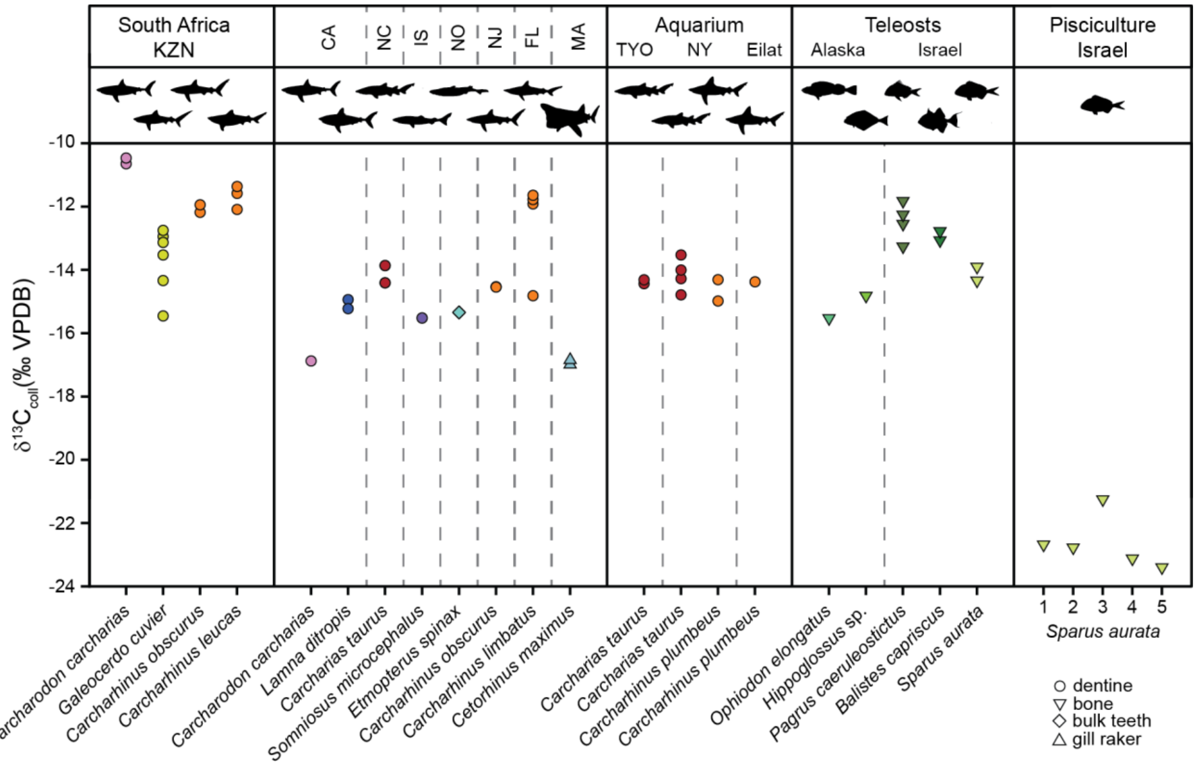

Supplementary Figure 3: Organic carbon isotope composition of extant elasmobranch and teleost fish teeth, bone, and gill raker. Specimens come from off the coast of KwaZulu-Natal (KZN) South Africa, California (CA), North Carolina (NC), Iceland (IS), Norway (NO), New Jersey (NJ), Florida (FL), Massachusetts (MA), Alaska and Israel. Aquarium sharks are from the Tokyo (TYO) and New York (NY) Aquariums and the Eilat (Israel) Underwater Observatory Park. Pisciculture *Sparus aurata* individuals are numbered and plotted individually to visualise the homogeneity among control fed individuals compared to wild elasmobranchs and teleosts. Samples are colour-coded following their genus, regardless of locality. Silhouettes are not to scale. Measurement uncertainty at the 1 SD level is smaller than the symbol size. Source data are provided as a Source Data file.

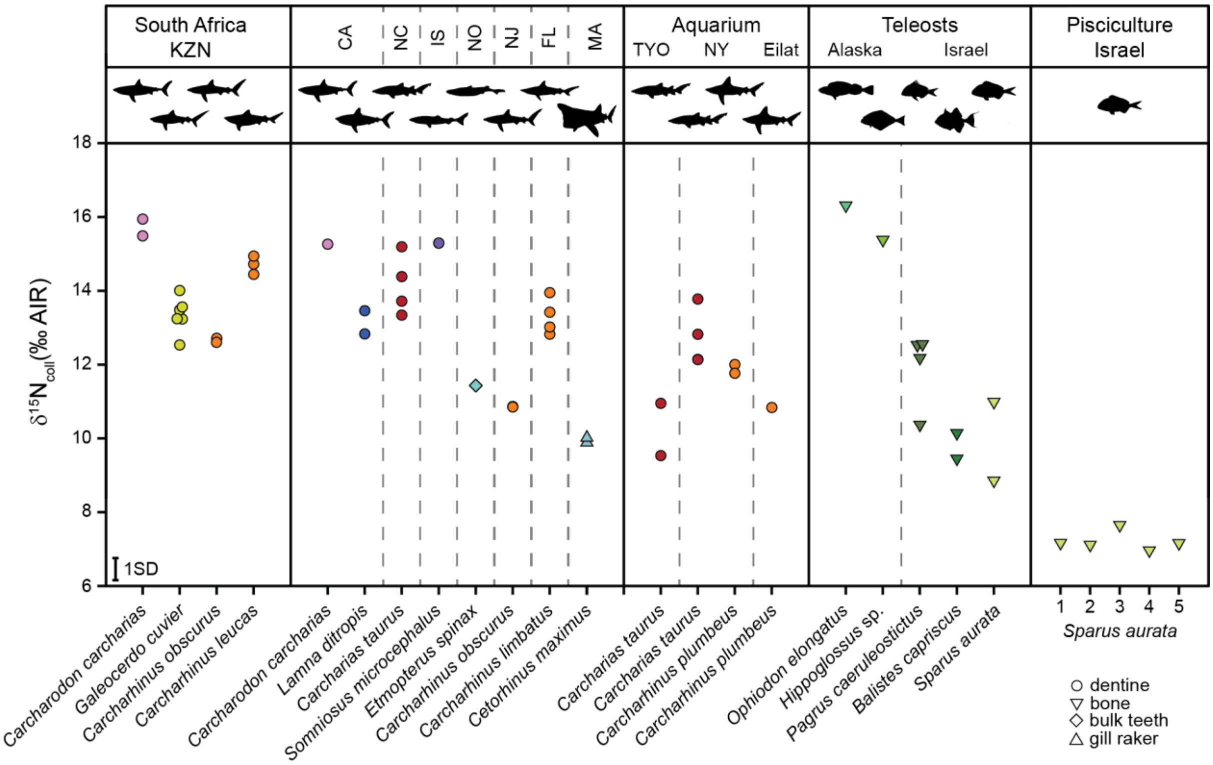

Supplementary Figure 4: Organic nitrogen isotope composition of extant elasmobranch and teleost fish teeth, bone, cartilage, and gill raker. Specimens come from off the coast of KwaZulu-Natal (KZN) South Africa, California (CA), North Carolina (NC), Iceland (IS), Norway (NO), New Jersey (NJ), Florida (FL), Massachusetts (MA), Alaska and Israel. Aquarium sharks are from the Tokyo (TYO) and New York (NY) Aquariums and the Eilat (Israel) Underwater Observatory Park. Pisciculture *Sparus aurata* individuals are numbered and plotted individually to visualise the homogeneity among control fed individuals compared to wild elasmobranchs and teleosts. Samples are colour-coded following their genus, regardless of locality. Silhouettes are not to scale. Measurement uncertainty is indicated at the 1 SD level. Source data are provided as a Source Data file.

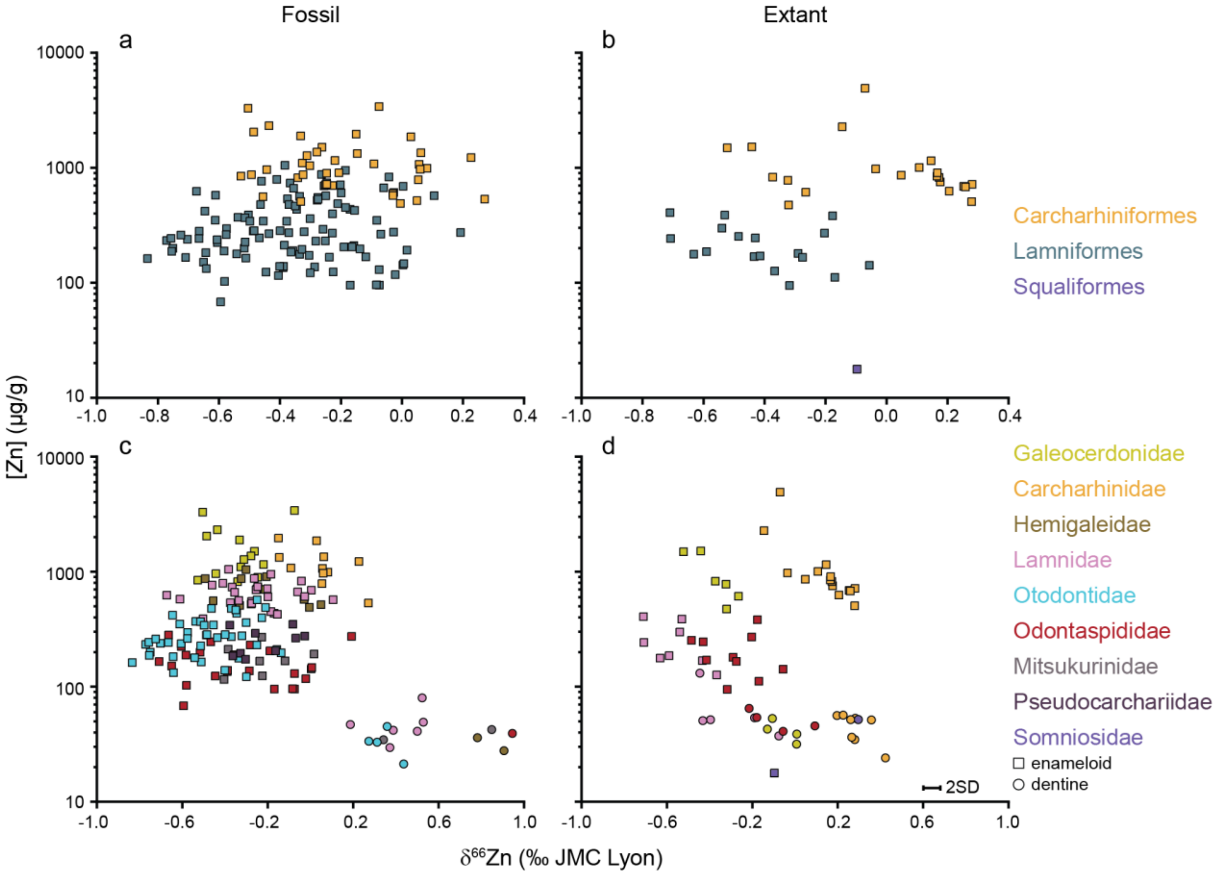

Supplementary Figure 5: Zinc isotope values versus Zn concentration ([Zn]) in fossil (a,c) and extant (b,d) elasmobranch enameloid. Samples are grouped and colour-coded by taxonomic order in (a) and (b) and after family in (c, d). (c) and (d) also include dentine. Measurement uncertainty is indicated at the 2 SD level. Source data are provided as a Source Data file.

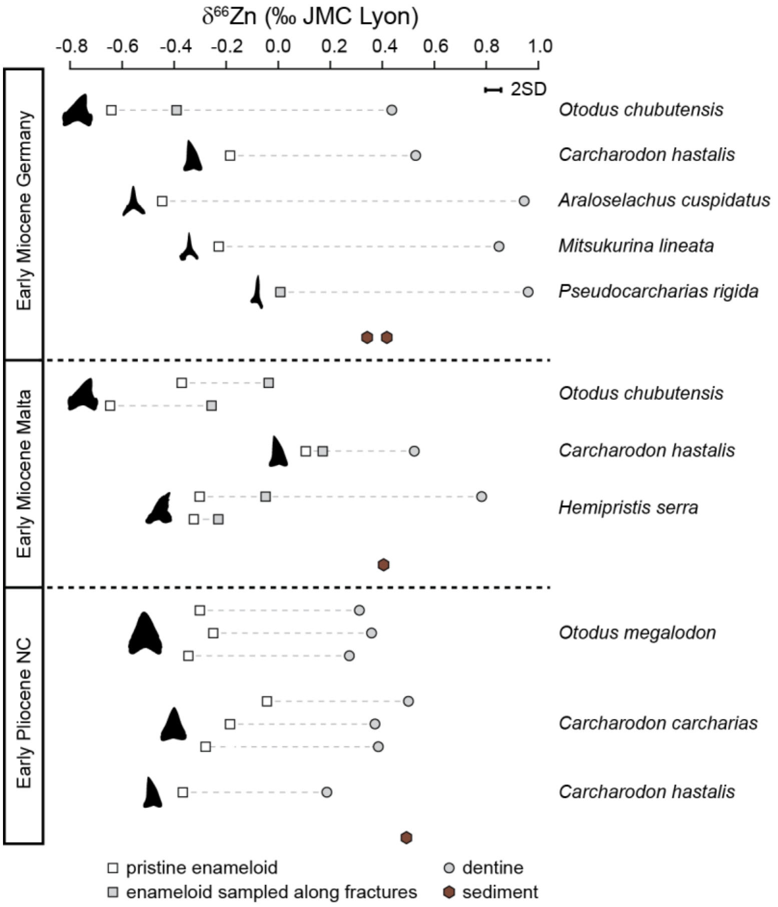

Supplementary Figure 6: Zinc isotope values of fossil elasmobranch tooth enameloid and dentine from the same tooth. Coloured squares indicate macroscopically pristine appearing enameloid, whereas the grey squares represent enameloid sampled along fractures. Note that the $\delta^{66}Zn$ difference between dentine (grey circles) and pristine appearing enameloid is significantly larger than the mean 0.2 ‰ difference between both dental tissues observed in extant teeth (Supplementary Figure 11). Carbonate sediment attached to individual teeth was also measured for these localities (brown hexagon). Measurement uncertainty is indicated at the 2 SD level. See also Supplementary Tables 1-2. Samples from North Carolina are indicated by (NC). Silhouettes are not to scale. Source data are provided as a Source Data file.

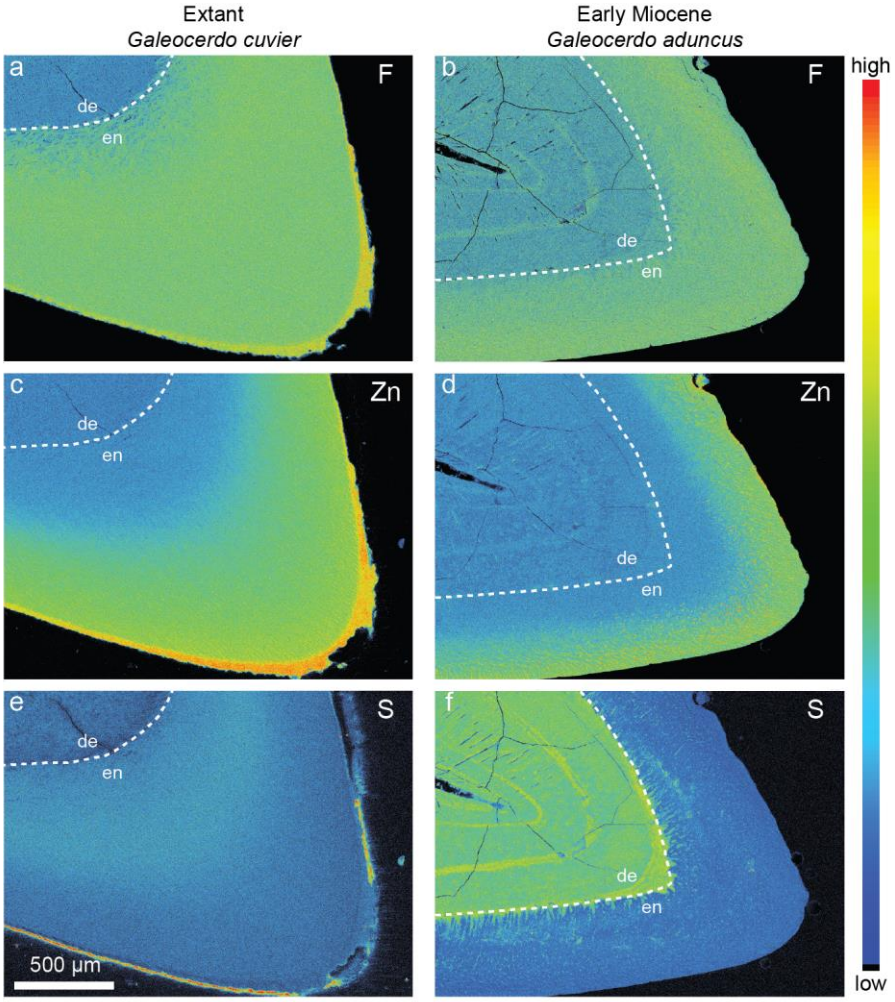

Supplementary Figure 7: Electron microprobe element distribution maps of a, c, e extant *Galeocerdo cuvier* (CMM-O-700, KZN) and b, d, f Early Miocene *Galeocerdo aduncus* (CMM-V-11539, Germany) teeth. a, b) Fluorine (F) distribution highlighting the higher content of fluorine in the enameloid (en) relative to the dentine (de) in both extant (a) and fossil (b) teeth. c, d) Zinc (Zn) distribution among the extant (c) and fossil (d) teeth demonstrating a significantly higher content in the outer enameloid only. Note that the fossil tooth also shows a Zn enrichment restricted to the outer enameloid. e, f) Sulphur (S) distribution in extant (e) and fossil (f) teeth. Note how S is enriched in the dentine of the fossil tooth only, indicating diagenetic incorporation of this element. The scale bar depicted in (e) applies to all panels. See section 'Methods' for analytical details.

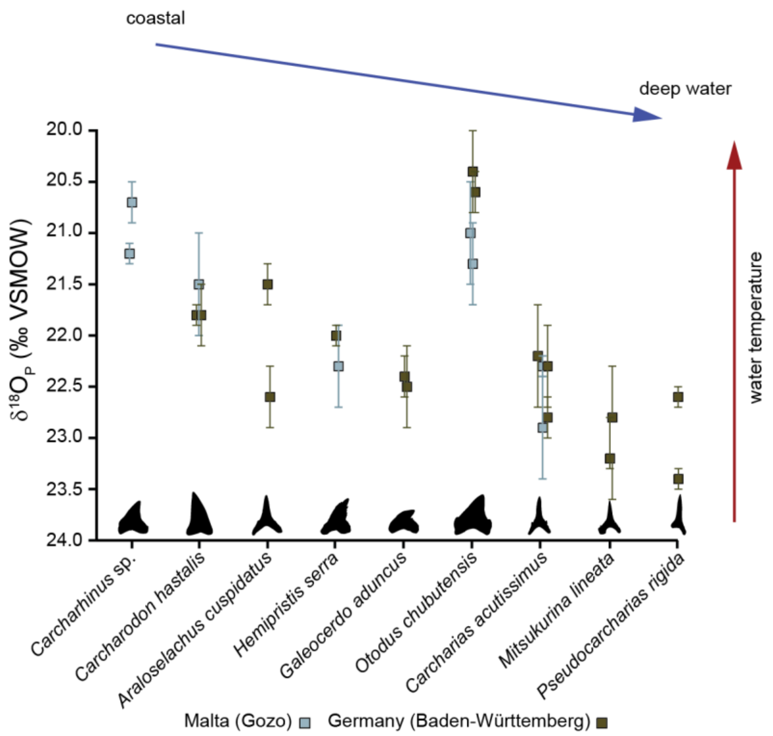

Supplementary Figure 8: Enameloid phosphate oxygen isotope ($\delta^{18}O_P$) values of selected European Early Miocene shark teeth. The y-axis is inverted. Note that higher $\delta^{18}O_P$ values (indicating lower water temperatures) are found in taxa for which modern representatives are commonly associated with more oceanic deep-water conditions (e.g., *Mitsukurina*, *Pseudocarcharias*). Lower $\delta^{18}O_P$ values, i.e., higher water temperature, are found in the typically coastal *Carcharhinus* species. The higher temperature indicated for *Otodus chubutensis* is likely related to mesothermy and elevated body temperature rather than environmental water temperature[90]. Individual points represent the mean of replicate TC/EA-IRMS measurements, with error bars corresponding to analytical precision defined as one standard deviation (1 SD) of sample replicate TC/EA-IRMS measurements. Each SD error bar is based on n = 3 independent IRMS measurements of a single silver phosphate preparation of the same biological sample. Silhouettes are not to scale. Source data are provided as a Source Data file.

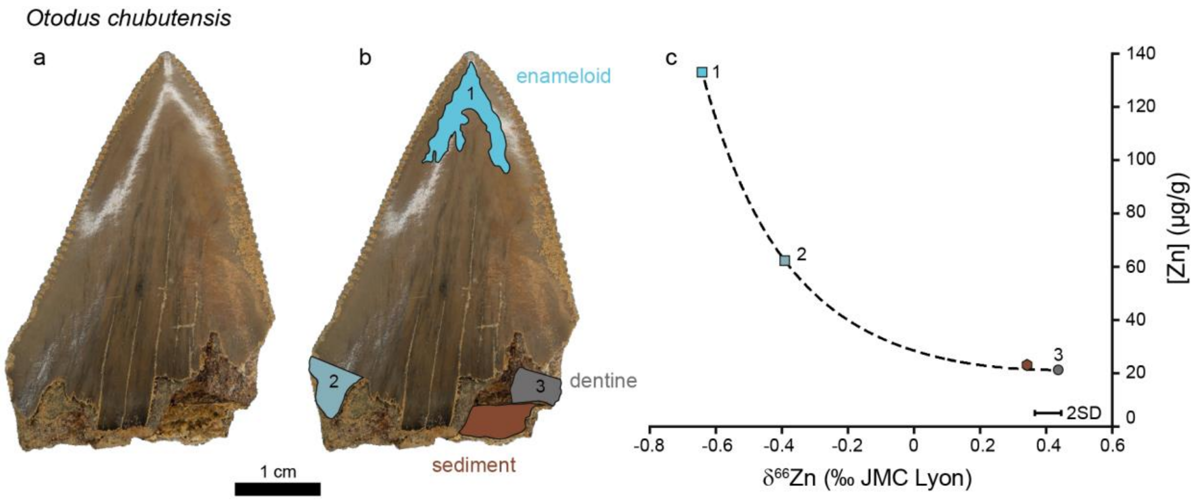

Supplementary Figure 9: Tooth of an Early Miocene *Otodus chubutensis* from Baden-Württemberg, southern Germany (a) with marked sample positions (b) and $\delta^{66}Zn$ values of this tooth (c). Pristine-appearing enameloid sampled at the apex (position 1) has a significantly lower $\delta^{66}Zn$ value and higher [Zn] than enameloid sampled along a fracture (position 2). Dentine in all analysed fossil teeth is always altered and has elevated $\delta^{66}Zn$ values, as shown here (position 3). Sediment collected from the specimen is also shown (brown hexagon). Measurement uncertainty is indicated at the 2 SD level. The dashed line represents the anticipated diagenetic trajectory. Source data are provided as a Source Data file.

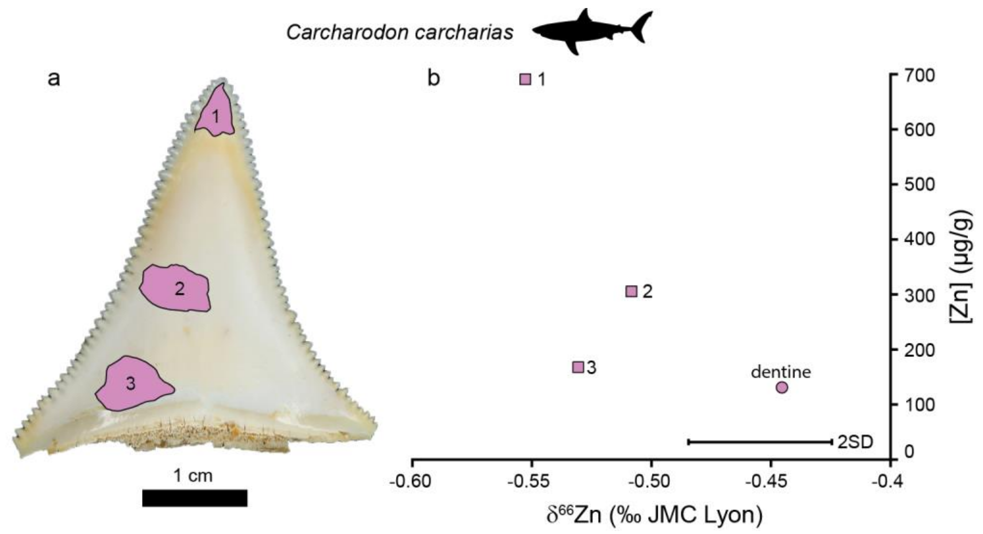

Supplementary Figure 10: Zinc isotope values versus Zn concentration ([Zn]) in an extant *Carcharodon carcharias* tooth (LACM 42728-1) enameloid (squares) and dentine (b). Enameloid sampling positions are numbered in (a). Note the intra-tooth variability in Zn concentration of the enameloid from apex to root while δ^{66}Zn shows no significant variability (Supplementary Table 3). Measurement uncertainty is indicated at the 2 SD level. Source data are provided as a Source Data file.

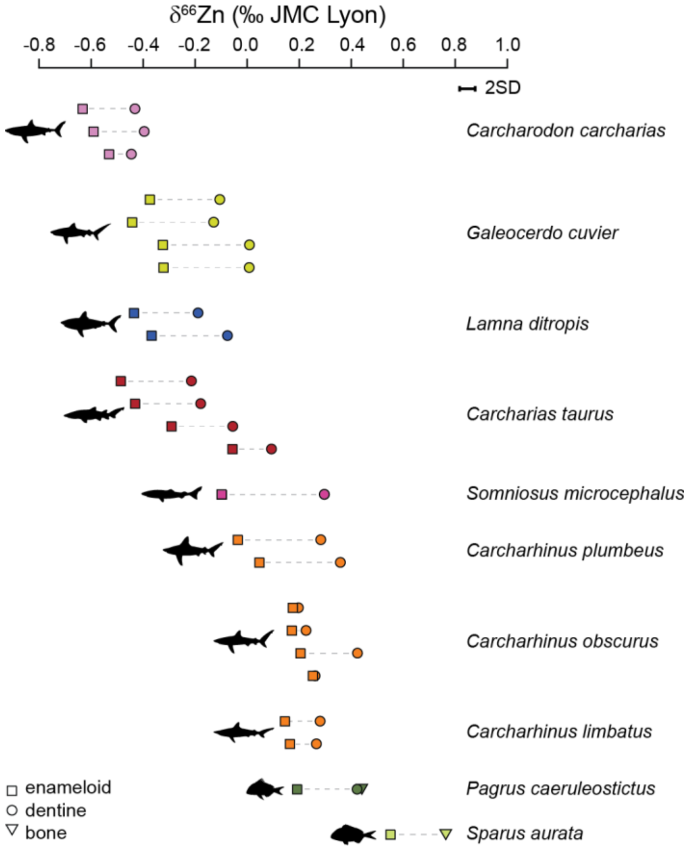

Supplementary Figure 11: Zinc isotope values of extant elasmobranch and teleost tooth enamel and dentine from the same tooth. For the two teleosts, enameloid is compared to bone from the same individual. Samples are colour-coded following their genus, regardless of locality. Measurement uncertainty is indicated at the 2 SD level. Silhouettes are not to scale. Source data are provided as a Source Data file.

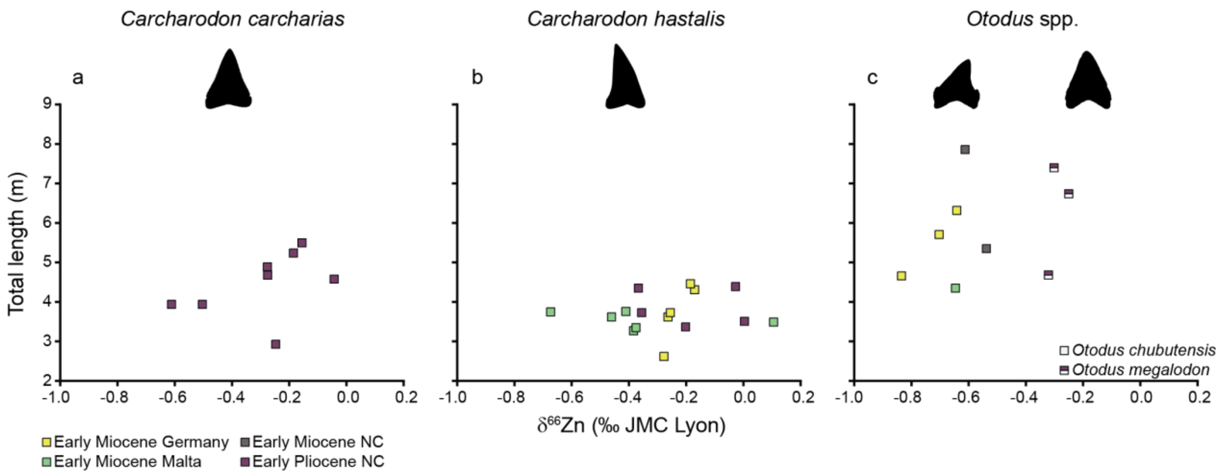

Supplementary Figure 12: Enameloid δ66Zn values versus total length for (a) *Carcharodon carcharias*, (b) *C. hastalis*, (c) *Otodus chubutensis* and *O. megalodon* inferred from tooth size. We observe no relevant correlation. Localities are colour-coded, yellow (Early Miocene, Germany), green (Early Miocene, Malta), grey (Early Miocene, North Carolina) and violet (Early Pliocene, North Carolina). Silhouettes are not to scale. Source data are provided as a Source Data file.

Supplementary Table 1: Zinc isotope values (in ‰ JMC Lyon) and Zn concentration from HCl-dissolvable sediment attached to individual teeth (see Supplementary Data 1). In the case of Malta (35793s), the sediment was attached to a tooth not analysed herein (n.a. indicates "not applicable").

Lab. ID	Tooth	Location	Sample size (mg)	δ^{66}Zn	δ^{67}Zn	δ^{68}Zn	Zn concentration (µg/g)
35787.3s	CMM-V-11563	Germany (Miocene)	46.64	0.34	0.70	0.75	23
35787.1s	CMM-V-11561	Germany (Miocene)	42.26	0.42	0.76	0.88	36
35793s	n.a.	Malta (Miocene)	28.21	0.41	0.68	0.85	33
35804.s	CMM-V-11647	North Carolina (Pliocene)	55.34	0.49	0.80	1.02	58

Supplementary Table 2: Zinc isotope values (in ‰ JMC Lyon) and Zn concentration from *Otodus chubutensis*, *Pseudocarcharias rigida*, *Carcharodon hastalis* and *Hemipristis serra* tooth enameloid sampled along fractures. For further sample information (see Supplementary Data 1 and Supplementary Figure 6).

Lab. ID	Tooth	Species	Location	Sample size (mg)	δ^{66}Zn	δ^{67}Zn	δ^{68}Zn	Zn concentration (µg/g)
35787.3e	CMM-V-11563	*O. chubutensis*	Germany (Miocene)	13.66	-0.39	-0.44	-0.66	62
35773.1e	CMM-V-11567	*P. rigida*	Germany (Miocene)	6.08	0.01	0.08	0.09	194
35791.1e	CMM-V-11601	*O. chubutensis*	Malta (Miocene)	8.82	-0.04	0.01	-0.01	50
35791.3e	CMM-V-11603	*O. chubutensis*	Malta (Miocene)	7.25	-0.26	-0.33	-0.45	80

35789.2e	CMM-V-11586	*C. hastalis*	Malta (Miocene)	11.84	0.17	0.33	0.37	193
35790.1e	CMM-V-11596	*H. serra*	Malta (Miocene)	5.65	-0.05	-0.08	-0.06	194
35790.2e	CMM-V-11597	*H. serra*	Malta (Miocene)	7.06	-0.23	-0.13	-0.37	195

Supplementary Table 3: Zinc isotope values (in ‰ JMC Lyon) and Zn concentration from a single extant *Carcharodon carcharias* tooth (LACM 42728-1 / laboratory ID 35847.1). The enameloid sample position is depicted in Supplementary Figure 10.

Tissue	Enameloid position	Sample size (mg)	δ^{66}Zn	δ^{67}Zn	δ^{68}Zn	Zn concentration (µg/g)
enameloid	3	11.94	-0.53	-0.74	-0.99	168
enameloid	2	12.24	-0.51	-0.73	-0.95	306
enameloid	1	11.71	-0.55	-0.77	-1.05	691
dentine	-	18.07	-0.45	-0.61	-0.81	131

Supplementary Table 4: Results from post-hoc Tukey pairwise comparisons of δ^{66}Zn values among species within the Germany (Miocene) assemblage (n = 59). Pairwise comparisons with statistically significant differences ($p < 0.05$) are identified with “ * ”.

Groups	Difference	Lower conf.	Upper conf.	Adj. *p*-value
Carcharias acutissimus - Araloselachus cuspidatus	0.365	0.069	0.661	0.006*
Carcharodon hastalis - Araloselachus cuspidatus	0.309	0.006	0.612	0.042*
Galeocerdo aduncus - Araloselachus cuspidatus	0.195	-0.101	0.491	0.440
Hemipristis serra - Araloselachus cuspidatus	0.376	0.080	0.672	0.004*
Mitsukurina lineata - Araloselachus cuspidatus	0.336	0.040	0.632	0.016*
Otodus chubutensis - Araloselachus cuspidatus	-0.109	-0.412	0.194	0.945
Pseudocarcharias rigida - Araloselachus cuspidatus	0.316	0.026	0.607	0.024*
Carcharodon hastalis - Carcharias acutissimus	-0.056	-0.306	0.195	0.997
Galeocerdo aduncus - Carcharias acutissimus	-0.170	-0.412	0.072	0.356
Hemipristis serra - Carcharias acutissimus	0.011	-0.230	0.253	1.000
Mitsukurina lineata - Carcharias acutissimus	-0.029	-0.270	0.213	1.000
Otodus chubutensis - Carcharias acutissimus	-0.474	-0.725	-0.224	$5.697E^{-6}$*
Pseudocarcharias rigida - Carcharias acutissimus	-0.049	-0.284	0.186	0.998

Galeocerdo aduncus* - *Carcharodon hastalis	-0.114	-0.365	0.136	0.833
Hemipristis serra* - *Carcharodon hastalis	0.067	-0.183	0.317	0.989
Mitsukurina lineata* - *Carcharodon hastalis	0.027	-0.223	0.277	1.000
Otodus chubutensis* - *Carcharodon hastalis	-0.419	-0.677	-0.160	$1.220E^{-4}$*
Pseudocarcharias rigida* - *Carcharodon hastalis	0.007	-0.237	0.250	1.000
Hemipristis serra* - *Galeocerdo aduncus	0.181	-0.060	0.423	0.278
Mitsukurina lineata* - *Galeocerdo aduncus	0.141	-0.100	0.383	0.593
Otodus chubutensis* - *Galeocerdo aduncus	-0.304	-0.555	-0.054	0.008*
Pseudocarcharias rigida* - *Galeocerdo aduncus	0.121	-0.114	0.356	0.731
Mitsukurina lineata* - *Hemipristis serra	-0.040	-0.282	0.202	0.999
Otodus chubutensis* - *Hemipristis serra	-0.486	-0.736	-0.235	$3.424E^{-6}$*
Pseudocarcharias rigida* - *Hemipristis serra	-0.060	-0.295	0.175	0.992
Otodus chubutensis* - *Mitsukurina lineata	-0.446	-0.696	-0.195	$2.072E^{-5}$*
Pseudocarcharias rigida* - *Mitsukurina lineata	-0.020	-0.255	0.215	1.000
Pseudocarcharias rigida* - *Otodus chubutensis	0.425	0.182	0.669	$3.046E^{-5}$*

Supplementary Table 5: Results from post-hoc Games-Howell pairwise comparisons among assemblages (indicated by their combined geographical location and their corresponding epoch) for δ^{66}Zn values of *Carcharhinus* spp. (n = 22). Pairwise comparisons with statistically significant differences ($p < 0.05$) are identified with " * ".

Groups	Estimate	Lower conf.	Upper conf.	Adj. p-value
***Carcharhinus* spp. (Miocene (Malta)) - *Carcharhinus* spp. (Pliocene (North Carolina))**	0.149	-0.133	0.430	0.307
***Carcharhinus* spp. (Miocene (Malta)) - *Carcharhinus* spp. (Miocene (North Carolina))**	0.119	-0.449	0.687	0.746
***Carcharhinus* spp. (Miocene (Malta)) - *Carcharhinus* spp. (Extant)**	0.233	0.044	0.422	0.022*
***Carcharhinus* spp. (Pliocene (North Carolina)) - *Carcharhinus* spp. (Miocene (North Carolina))**	-0.030	-0.590	0.530	0.993
***Carcharhinus* spp. (Pliocene (North Carolina)) - *Carcharhinus* spp. (Extant)**	0.084	-0.259	0.427	0.580
***Carcharhinus* spp. (Miocene (North Carolina)) - *Carcharhinus* spp. (Extant)**	0.114	-0.574	0.802	0.731

Supplementary Table 6: Results from post-hoc Tukey pairwise comparisons of δ^{66}Zn values among species within the North Carolina (Miocene) assemblage (n = 13). Pairwise comparisons with statistically significant differences ($p < 0.05$) are identified with " * ".

Groups	Difference	Lower conf.	Upper conf.	Adj. p-value
***Carcharias* sp. - *Carcharhinus* sp.**	-0.420	-1.040	0.200	0.219
***Carcharias taurus* - *Carcharhinus* sp.**	-0.390	-1.010	0.230	0.269
***Otodus chubutensis* - *Carcharhinus* sp.**	-0.674	-1.254	-0.094	0.023*
***Carcharias taurus* - *Carcharias* sp.**	0.030	-0.590	0.650	0.999
***Otodus chubutensis* - *Carcharias* sp.**	-0.254	-0.834	0.326	0.547
Otodus chubutensis* - *Carcharias taurus	-0.284	-0.864	0.296	0.460

Supplementary Table 7: Results from post-hoc Games-Howell pairwise comparisons of δ^{66}Zn values among species within the North Carolina (Pliocene) assemblage (n = 13). Pairwise comparisons with statistically significant differences ($p < 0.05$) are identified with " * ".

Groups	Estimate	Lower conf.	Upper conf.	Adj. p-value
Carcharhinus* sp. - *Carcharodon carcharias	-0.405	-0.689	-0.122	0.009*
Carcharhinus* sp. - *Carcharodon hastalis	-0.310	-0.602	-0.018	0.039*
Carcharhinus* sp. - *Otodus megalodon	-0.500	-0.775	-0.226	0.006*
Carcharodon carcharias* - *Carcharodon hastalis	0.095	-0.177	0.368	0.728
Carcharodon carcharias* - *Otodus megalodon	-0.095	-0.321	0.131	0.611
Carcharodon hastalis* - *Otodus megalodon	-0.190	-0.426	0.045	0.122

Supplementary Table 8: Results from post-hoc Games-Howell pairwise comparisons of δ^{66}Zn values among species within the Malta (Miocene) assemblage (n = 30). Pairwise comparisons with statistically significant differences ($p < 0.05$) are identified with " * ".

Groups	Estimate	Lower conf.	Upper conf.	Adj. p-value
Carcharhinus* sp. - *Carcharias acutissimus	-0.528	-0.827	-0.229	0.003*
Carcharhinus* sp. - *Carcharodon hastalis	-0.333	-0.772	0.106	0.152
Carcharhinus* sp. - *Galeocerdo aduncus	-0.348	-0.635	-0.061	0.020*
Carcharhinus* sp. - *Hemipristis serra	-0.338	-0.684	0.008	0.054
Carcharhinus* sp. - *Otodus chubutensis	-0.456	-0.751	-0.160	0.003*
Carcharias acutissimus* - *Carcharodon hastalis	0.195	-0.254	0.644	0.611
Carcharias acutissimus* - *Galeocerdo aduncus	0.180	-0.144	0.504	0.348

Carcharias acutissimus - Hemipristis serra	0.190	-0.178	0.558	0.357
Carcharias acutissimus - Otodus chubutensis	0.073	-0.253	0.398	0.964
Carcharodon hastalis - Galeocerdo aduncus	-0.015	-0.461	0.431	1.000
Carcharodon hastalis - Hemipristis serra	-0.005	-0.464	0.454	1.000
Carcharodon hastalis - Otodus chubutensis	-0.123	-0.570	0.325	0.919
Galeocerdo aduncus - Hemipristis serra	0.010	-0.352	0.372	1.000
Galeocerdo aduncus - Otodus chubutensis	-0.108	-0.425	0.210	0.834
Hemipristis serra - Otodus chubutensis	-0.118	-0.466	0.231	0.799

Supplementary Table 9: Results from post-hoc Games-Howell pairwise comparisons among assemblages (indicated by their combined geographical location and their corresponding epoch) for $\delta^{66}Zn$ values of *Carcharodon* spp. (n = 31). Pairwise comparisons with statistically significant differences ($p < 0.05$) are identified with " * ".

Groups	Estimate	Lower conf.	Upper conf.	Adj. *p*-value
C. carcharias **(Extant) -** ***C. hastalis*** **(Miocene (Malta))**	0.208	-0.209	0.624	0.400
C. carcharias **(Extant) -** ***C. carcharias*** **(Pliocene (North Carolina))**	0.284	0.049	0.519	0.019*
C. carcharias **(Extant) -** ***C. hastalis*** **(Miocene (Germany))**	0.347	0.202	0.492	$1.590E^{-4}$*
C. carcharias **(Extant) -** ***C. hastalis*** **(Pliocene (North Carolina))**	0.379	0.127	0.632	0.007*
C. hastalis **(Miocene (Malta)) -** ***C. carcharias*** **(Pliocene (North Carolina))**	0.076	-0.342	0.495	0.968
C. hastalis **(Miocene (Malta)) -** ***C. hastalis*** **(Miocene (Germany))**	0.139	-0.273	0.551	0.725
C. hastalis **(Miocene (Malta)) -** ***C.hastalis*** **(Pliocene (North Carolina))**	0.172	-0.250	0.593	0.646
C. carcharias **(Pliocene (North Carolina)) -** ***C. hastalis*** **(Miocene (Germany))**	0.063	-0.179	0.305	0.910
C. carcharias **(Pliocene (North Carolina)) -** ***C. hastalis*** **(Pliocene (North Carolina))**	0.095	-0.197	0.388	0.830
C. hastalis **(Miocene (Germany)) -** ***C. hastalis*** **(Pliocene (North Carolina))**	0.032	-0.222	0.286	0.991

Supplementary Table 10: Results from post-hoc Games-Howell pairwise comparisons among assemblages (indicated by their combined geographical location and their corresponding epoch) for $\delta^{66}Zn$ values of *Otodus* spp. (n = 42). Pairwise comparisons with statistically significant differences ($p < 0.05$) are identified with " * ".

Groups	Estimate	Lower conf.	Upper conf.	Adj. p-value
***O. chubutensis* (Miocene (Germany)) - *O. chubutensis* (Miocene (Malta))**	0.157	-0.144	0.458	0.536
***O. chubutensis* (Miocene (Germany)) - *O. chubutensis* (Miocene (North Carolina))**	0.057	-0.275	0.389	0.982
***O. chubutensis* (Miocene (Germany)) - *O. megalodon* (Miocene-Pliocene (Florida))**	0.304	0.056	0.553	0.014*
***O. chubutensis* (Miocene (Germany)) - *O. megalodon* (Pliocene (Japan))**	0.022	-0.242	0.287	1.000
***O. chubutensis* (Miocene (Germany)) - *O. chubutensis* (Miocene (North Carolina))**	0.261	0.032	0.489	0.023*
***O. chubutensis* (Miocene (Malta)) - *O. megalodon* (Miocene-Pliocene (Florida))**	-0.100	-0.451	0.251	0.900
***O. chubutensis* (Miocene (Malta)) - *O. megalodon* (Pliocene (Japan))**	0.148	-0.144	0.439	0.555
***O. chubutensis* (Miocene (Malta)) - *O. megalodon* (Pliocene (North Carolina))**	-0.135	-0.436	0.167	0.660
***O. chubutensis* (Miocene (Malta)) - *O. megalodon* (Pliocene (North Carolina))**	0.104	-0.174	0.382	0.794
***O. chubutensis* (Miocene (North Carolina)) - *O. megalodon* (Miocene-Pliocene (Florida))**	0.248	-0.081	0.576	0.148
***O. chubutensis* (Miocene (North Carolina)) - *O. megalodon* (Pliocene (Japan))**	-0.035	-0.372	0.303	0.998
***O. chubutensis* (Miocene (North Carolina)) - *O. megalodon* (Pliocene (North Carolina))**	0.204	-0.123	0.530	0.240
***O. megalodon* (Miocene-Pliocene (Florida)) - *O. megalodon* (Pliocene (Japan))**	-0.282	-0.534	-0.030	0.027*
***O. megalodon* (Miocene-Pliocene (Florida)) - *O. megalodon* (Pliocene (North Carolina))**	-0.044	-0.252	0.165	0.979
***O. megalodon* (Pliocene (Japan)) - *O. megalodon* (Pliocene (North Carolina))**	0.238	0.005	0.472	0.045*

Supplementary Table 11: Results from post-hoc Games-Howell pairwise comparisons of $\delta^{66}Zn$ values between *Otodus* spp. and *Carcharodon* spp. (n = 73). Pairwise comparisons with statistically significant differences ($p < 0.05$) are identified with " * ".

Groups	Estimate	Lower conf.	Upper conf.	Adj. p-value
***O. chubutensis* (Fossil) - *C. carcharias* (Extant)**	-0.006	-0.147	0.134	1.000
***O. chubutensis* (Fossil) - *O. megalodon* (Fossil)**	0.144	-0.008	0.296	0.070
***O. chubutensis* (Fossil) - *C. carcharias* (Fossil)**	0.278	0.035	0.520	0.022*
***O. chubutensis* (Fossil) - *C. hastalis* (Fossil)**	0.307	0.139	0.475	$6.780E^{-5}$*
***C. carcharias* (Extant) - *O. megalodon* (Fossil)**	0.150	0.026	0.275	0.014*
***C. carcharias* (Extant) - *C. carcharias* (Fossil)**	0.284	0.049	0.519	0.019*
***C. carcharias* (Extant) - *C. hastalis* (Fossil)**	0.313	0.169	0.457	$2.390E^{-5}$*
***O. megalodon* (Fossil) - *C. carcharias* (Fossil)**	0.133	-0.104	0.371	0.412
***O. megalodon* (Fossil) - *C. hastalis* (Fossil)**	0.163	0.007	0.318	0.037*
***C. carcharias* (Fossil) - *C. hastalis* (Fossil)**	0.029	-0.215	0.273	0.995

Supplementary Table 12: Results from post-hoc Games-Howell pairwise comparisons of $\delta^{66}Zn$ values between Atlantic and Tethys/Paratethys *Otodus* spp. and *Carcharodon* spp. excluding the Japanese (Pacific) population (n = 68). Pairwise comparisons with statistically significant differences ($p < 0.05$) are identified with " * ".

Groups	Estimate	Lower conf.	Upper conf.	Adj. p-value
***C. carcharias* (Extant) - *C. carcharias* (Fossil)**	0.284	0.049	0.519	0.019*
***C. carcharias* (Extant) - *C. hastalis* (Fossil)**	0.313	0.169	0.457	$2.390E^{-5}$*
***C. carcharias* (Extant) - *O. chubutensis* (Fossil)**	0.006	-0.134	0.147	1.000
***C. carcharias* (Extant) - *O. megalodon* (Fossil)**	0.206	0.088	0.324	$6.790E^{-4}$*
***C. carcharias* (Fossil) - *C. hastalis* (Fossil)**	0.029	-0.215	0.273	0.995
***C. carcharias* (Fossil) - *O. chubutensis* (Fossil)**	-0.278	-0.520	-0.035	0.022*
***C. carcharias* (Fossil) - *O. megalodon* (Fossil)**	-0.078	-0.314	0.158	0.810
***C. hastalis* (Fossil) - *O. chubutensis* (Fossil)**	-0.307	-0.475	-0.139	$6.780E^{-5}$*
***C. hastalis* (Fossil) - *O. megalodon* (Fossil)**	-0.107	-0.257	0.043	0.260
***O. chubutensis* (Fossil) - *O. megalodon* (Fossil)**	0.200	0.054	0.346	0.003*

Supplementary References

1. Ben-David, M. & Flaherty, E. A. Stable isotopes in mammalian research: a beginner's guide. *J. Mammal.* **93,** 312-328 (2012).
2. Clementz, M. T. New insight from old bones: stable isotope analysis of fossil mammals. *J. Mammal.* **93,** 368-380 (2012).
3. Heuser, A., Tütken, T., Gussone, N. & Galer, S. J. Calcium isotopes in fossil bones and teeth—Diagenetic versus biogenic origin. *Geochim. Cosmochim. Acta* **75,** 3419-3433 (2011).
4. Jaouen, K., Beasley, M., Schoeninger, M., Hublin, J. J. & Richards, M. P. Zinc isotope ratios of bones and teeth as new dietary indicators: results from a modern food web (Koobi Fora, Kenya). *Sci. Rep.* **6,** 26281 (2016). https://doi.org/10.1038/srep26281
5. Bourgon, N. et al. Zinc isotopes in Late Pleistocene fossil teeth from a Southeast Asian cave setting preserve paleodietary information. *Proc. Natl. Acad. Sci.* **117,** 4675-4681 (2020).
6. Martin, J. E., Tacail, T., Adnet, S., Girard, C. & Balter, V. Calcium isotopes reveal the trophic position of extant and fossil elasmobranchs. *Chem. Geol*. **415,** 118-125 (2015).
7. Jaouen, K., Szpak, P. & Richards, M. P. Zinc isotope ratios as indicators of diet and trophic level in Arctic marine mammals. *PLoS ONE* **11,** (2016). https://doi.org/10.1371/journal.pone.0152299
8. McCormack, J. et al. Zinc isotopes from archaeological bones provide reliable trophic level information for marine mammals. *Commun. Biol.* **4,** 683 (2021). https://doi.org/10.1038/s42003-021-02212-z
9. Balter, V. et al. Contrasting Cu, Fe, and Zn isotopic patterns in organs and body fluids of mice and sheep, with emphasis on cellular fractionation. *Metallomics* **5,** 1470-1482 (2013).
10. Moynier, F., Fujii, T., Shaw, A. S., & Le Borgne, M. Heterogeneous distribution of natural zinc isotopes in mice. *Metallomics* **5,** 693-699 (2013).
11. Mahan, B., Moynier, F., Jørgensen, A. L., Habekost, M., & Siebert, J. Examining the homeostatic distribution of metals and Zn isotopes in Göttingen minipigs. *Metallomics* **10,** 1264-1281 (2018).
12. Jaouen, K. et al. Dynamic homeostasis modeling of Zn isotope ratios in the human body. *Metallomics* **11,** 1049-1059 (2019).
13. Bourgon, N. et al. Diet of a Late Pleistocene early modern human from Southeast Asia inferred from zinc and carbon isotopes. *J. Hum. Evol.* **161,** 103075 (2021). https://doi.org/10.1016/j.jhevol.2021.103075
14. Sieber, M. et al. Cycling of zinc and its isotopes across multiple zones of the Southern Ocean: Insights from the Antarctic Circumnavigation Expedition. *Geochim. Cosmochim. Acta* **268**, 310-324 (2020).
15. Samanta, M., Ellwood, M. J., Sinoir, M. & Hassler, C. S. Dissolved zinc isotope cycling in the Tasman Sea, SW Pacific Ocean. *Mar. Chem.* **192,** 1-12 (2017).
16. Cloquet, C., Carignan, J., Lehmann, M. F., & Vanhaecke, F. Variation in the isotopic composition of zinc in the natural environment and the use of zinc isotopes in biogeosciences: a review. *Anal. Bioanal. Chem.* **390,** 451-463 (2008).
17. Little, S. H., Vance, D., Walker-Brown, C. & Landing, W. M. The oceanic mass balance of copper and zinc isotopes, investigated by analysis of their inputs, and outputs to ferromanganese oxide sediments. *Geochim. Cosmochim. Acta* **125,** 673-693 (2014).
18. John, S. G. & Conway, T. M. A role for scavenging in the marine biogeochemical cycling of zinc and zinc isotopes. *Earth Planet. Sci. Lett.* **394,** 159-167 (2014).
19. Conway, T. M. & John, S. G. The biogeochemical cycling of zinc and zinc isotopes in the North Atlantic Ocean. *Global Biogeochem. Cycles* **28,** 1111-1128 (2014).
20. Wyatt, N. J. et al. Biogeochemical cycling of dissolved zinc along the GEOTRACES South Atlantic transect GA10 at 40°S, *Global Biogeochem. Cycles* **28,** 44–56 (2014).

21. Liao, W. H. et al. Zn isotope composition in the water column of the Northwestern Pacific Ocean: the importance of external sources. *Global Biogeochem. Cycles* **34,** e2019GB006379 (2020). https://doi.org/10.1029/2019GB006379
22. Maréchal, C. N., Nicolas, E., Douchet, C. & Albarède, F. Abundance of zinc isotopes as a marine biogeochemical tracer. *Geochem. Geophys. Geosyst.* **1,** 1015 (2000).
23. John, S. G. *The Marine Biogeochemistry of Zinc Isotopes*. [Doctoral Thesis]. (Massachusetts Institute of Technology, 2007).
24. von Doppler, G., Heissig, K. & Reichenbacher, B. Die Gliederung des Tertiärs im süddeutschen Molassebecken. *Newsl. Stratigr.*, **41,** 359-375 (2005).
25. Heckeberg N., Pippèrr M., Läuchli B., Heimann F.U.M. & Reichenbacher B. The Upper Marine Molasse (Burdigalian, Ottnangian) in Southwest Germany – facies interpretation and a new lithostratigraphic terminology. *Z. Dtsch. Ges. Geowiss*. **161,** 285-302 (2010).
26. Heimann, F.U.M., Schmid, D.U., Pippèrr, M. & Reichenbacher, B. Re-interpreting the Baltringen Horizont as a subtidal channel facies: Implications for a new understanding of the Upper Marine Molasse «Cycles» (Early Miocene). *N. Jb. Geol. Palaeont. Abh.* **254,** 135-149 (2009).
27. Probst, J. Beschreibung einiger Lokalitäten in der Molasse von Oberschwaben. *Jahresh. Ver. vaterl. Naturkd. Württemb.* **44,** 64-114 (1888).
28. Pippèrr M. Characterisation of Ottnangian palaeoenvironments in the North Alpine Foreland Basin using benthic foraminifera – a review on the Upper Marine Molasse of Southern Germany. *Mar. Micropaleontol.* **79,** 80-99 (2011).
29. Barthelt, D., Fejfar, O., Pfeil, F.H. & Unger, E. Notizen zu einem Profil der Selachier- Fundstelle Walbertsweiler im Bereich der miozänen Oberen Meeresmolasse Süddeutschlands. *Münch. Geowiss. Abh. A* **19,** 195-208 (1991).
30. Probst, J. Beiträge zur Kenntniss der fossilen Fische aus der Molasse von Baltringen. II. Batoidei A. Günther. Klein- und grosszahnige Rochen. *Jahresh. Ver. vaterl. Naturkd. Württemb.* **33,** 69-103 (1877).
31. Probst, J. Beiträge zur Kenntniss der fossilen Fische aus der Molasse von Baltringen. Hayfische (Selachoidei A. Günther). *Jahresh. Ver. vaterl. Naturkd. Württemb.* **34,** 113-154 (1878).
32. Probst, J. Beiträge zur Kenntniss der fossilen Fische aus der Molasse von Baltringen. Hayfische (Selachoidei A. Günther) (Schluss). *Jahresh. Ver. vaterl. Naturkd. Württemb.* **35,** 127-191 (1879).
33. Martinelli, M., Bistacchi, A., Balsamo, F. & Meda, M. Late Oligocene to Pliocene extension in the Maltese Islands and implications for geodynamics of the Pantelleria Rift and Pelagian Platform. *Tectonics* **38,** 3394-3415 (2019).
34. Pedley, H. M., House, M.R. & Waugh, B. The geology of Malta and Gozo. *Proc. Geol. Assoc*. **87,** 325-341 (1976).
35. Foresi, L. M., Mazzei, R., Salvatorini, G. & Donia, F. Biostratigraphy and chronostratigraphy of the Maltese Lower Globigerina Limestone Member (Globigerina Limestone Formation): new preliminary data based on calcareous plankton. *Boll. Soc. Paleontol. Ital.* **46,** 175-181 (2008).
36. Janssen, A. W. Systematics and biostratigraphy of holoplanktonic Mollusca from the Oligo-Miocene of the Maltese Archipelago. *Boll. Mus. Regionale. Sci. Nat. Torino* **28,** 197-601 (2012).
37. Menesini, E. Ittiodontoliti delle formazioni terziaire dell' arcipelago maltese. *Palaeontogr. Ital.* **67,** 121-161 (1974).
38. Ward, D. J. & Bonavia, C. G. Additions to and a review of the Miocene shark and ray fauna of Malta. *The Central Mediterranean Naturalist* **3,** 131-146 (2001).
39. Snyder, S. W., Hine, A. C. & Riggs, S. Miocene Seismic Stratigraphy, Structural Framework, and Sea-Level Cyclicity: North Carolina Continental Shelf. *Southeast. Geol.* **23,** 247-266 (1982).

40. Riggs, S., York, L., Wehmiller, J. & Snyder S. W. Depositional patterns resulting from high-frequency Quaternary sea-level fluctuations in northeastern North Carolina. *SEPM Spec. Publ.* **48,** 142-153 (1992).
41. Horton, B.W. et al. Holocene sea-level changes along the North Carolina coastline and their implications for glacial isostatic adjustment models. *Quat. Sci. Rev.* 28, 1725-1736 (2009).
42. Harris, M. et al. Continental shelf landscapes of the southeastern United States since the last interglacial. *Geomorphology* **203,** 6-24 (2013).
43. Lewis, D. et al. *Preliminary stratigraphic report on the Pungo River Formation in Onslow Bay, continental shelf, North Carolina*. In: *Miocene Symposium of the Southeastern United States* (ed Scott, T.) 122-137 (Florida Bureau of Geology, Special publication, 1982).
44. Riggs, S. & Mallette., P. *Patterns of phosphate deposition and lithofacics relationships within the Miocene Pungo River Formation, North Carolina continental margin*. In: *Phosphoritc Deposits of the World, Volume III: Neogene to Modern phosphorites* (eds Burnett, W. & Riggs, S.) 424-443 (Cambridge University Press, Cambridge, UK, 1990).
45. Maisch, H., IV, Becker, M. A. & Chamberlain J. A. Jr. Lamniform and Carcharhiniform sharks from the Pungo River and Yorktown formations (Miocene-Pliocene) from the submerged continental shelf, Onslow Bay, North Carolina. *Copeia* **106,** 353-374 (2018).
46. Maisch, H., IV, Becker, M. A. & Chamberlain J. A. Jr. Macroborings in *Otodus megalodon* and *Otodus chubutensis* shark teeth from the submerged shelf of Onslow Bay, North Carolina, USA: Implications for processes of lag deposit formation. *Ichnos* **27,** 122-141 (2019).
47. Riggs, S., Lewis, D., Scarborough, A. & Snyder, S. W. Cyclic deposition of Neogene phosphorites in the Aurora Area, North Carolina, and their possible relationship to global sea-level fluctuations. *Southeast. Geol.* **23,** 189-204 (1982).
48. Purdy, R. et al. *The Neogene sharks, rays, and bony fish from Lee Creek Mine, Aurora, North Carolina*. In: *Geology and Paleontology of the Lee Creek Mine, North Carolina, III. Smithsonian Contributions to Paleobiology* 90, (eds Ray, C. & Bohaska, D.) 71-202 (Smithsonian Institution Press, Washington D.C., 2001).
49. Ward, L. *Synthesis of Paleontological and Stratigraphic Investigations at the Lee Creek Mine, Aurora, NC (1958-2007)*. In: *Carolina Geological Society Fieldtrip guidebook, 2007*. (ed Ward, L.) 1-138 (*Virginia Museum of Natural History Guidebook* 8, 2007).
50. Maisch, H., IV, Becker, M. A. & Chamberlain J. A. Jr. Chondrichthyans from a lag deposit between the Shark River Formation (Middle Eocene) and Kirkwood Formation (Early Miocene), Monmouth County, New Jersey. *Paludicola* **10,** 149-183 (2015).
51. Hastings, A. & Dooley A. Jr. *Fossil-collecting from the middle Miocene Carmel Church Quarry marine ecosystem in Carolina County, Virginia*, In: *Geological Society of America Field Guide 47: From the Blue Ridge to the beach: Geological Field Excursions across Virginia* (eds Bailey, C., Jaye, S.) 77-88 (Geological Society of America, Virginia, 2017).
52. Kent, B. *The cartilaginous fishes (chimaeras, sharks and rays) of Calvert Cliffs, Maryland, USA*. In: The Geology and Vertebrate Paleontology of Calvert Cliffs, Smithsonian Contributions to Paleobiology, 100. (ed Godfrey, S.) 45-160 (Smithsonian Institution Scholarly Press, 2018).
53. Scott, T. et al. *Geologic map of the State of Florida*. Florida Geological Survey (2001).
54. Cunningham, K. J. et al. Interplay of Late Cenozoic siliciclastic supply and carbonate response on the southeast Florida platform. *J. Sediment. Res.* **73,** 31-46 (2003).
55. Fuqua, R., *Hunting Fossil Shark Teeth in Venice, Florida: The Complete Guide: on the Beach, SCUBA Diving, and Inland*. (CreateSpace Independent Publishing Platform, South Carolina, 2011)
56. Bryan, J., Scott, T. & Means, G. *Roadside Geology of Florida* (Mountain Press Publishing Company, Missoula, Montana, 2014).
57. Scott, T. M. The lithostratigraphy of the Hawthorn Group (Miocene) of Florida. *Florida Geol. Surv. Bull.* 59 (1988).
58. Scott, T. M. 1990. The lithostratigraphy of the Hawthorn Group of peninsular Florida. *Open File Report Florida Geol. Surv.* 36 (1990).

59. Perez, V. J. & Marks, K. W., The first documented fossil records of *Isistius* and *Squatina* (Chondrichthyes) from Florida, with an overview of the associated vertebrate fauna. *Bull. Florida Museum Nat. Hist*. **55,** 139-155 (2017).
60. Riggs, S. R., Phosphorite sedimentation in Florida; a model phosphogenic system. *Econ. Geol*. **74,** 285-314 (1979).
61. Riggs, S. R. Intraclast and pellet phosphorite sedimentation in the Miocene of Florida. *J. Geol. Soc*. **137,** 741-748 (1980).
62. Hulbert, R. *The Fossil Vertebrates of Florida* (University Press of Florida, Gainesville, 2001).
63. Itoigawa, J. et al. *Carcharodon carcharias* (Linne) shark teeth from the Pliocene Na-arai Formation, Choshi Peninsula, environs of Tokyo, Japan. *Bull. Mizunami Fossil Mus.* **2,** 91-102 (1975).
64. Oishi, M. & Hasegawa, Y. Diversity of Pliocene mysticetes from eastern Japan. *Isl. Arc* **3,** 346-452 (1995).
65. Boessenecker, R. W. et al. The Early Pliocene extinction of the mega-toothed shark *Otodus megalodon*: a view from the eastern North Pacific. *PeerJ* **7,** e6088 (2019). https://doi.org/10.7717/peerj.6088
66. Dettman, D. L. et al. Seasonal stable isotope evidence for a strong Asian monsoon. *Geology* **29,** 31-34. (2001).
67. Tütken, T., Vennemann, T. W., Janz, H. & Heizmann, E. P. J. Palaeoenvironment and palaeoclimate of the Middle Miocene lake in the Steinheim basin, SW Germany: A reconstruction from C, O, and Sr isotopes of fossil remains. *Palaeogeogr. Palaeoclimatol. Palaeoecol.* **241,** 457-491. (2006).
68. Enax, J., Prymak, O., Raabe, D. & Epple, M. Structure, composition, and mechanical properties of shark teeth. *J. Struct. Biol.* **178,** 290-299 (2012).
69. Brand, W. A. et al. Comprehensive inter-laboratory calibration of reference materials for $\delta^{18}O$ versus VSMOW using various on-line high-temperature conversion techniques. *Rapid Commun. Mass Spectrom.* **23,** 999-1019 (2009).
70. Froese, R. & Pauly, D. FishBase. World Wide Web electronic publication. https://www.fishbase.se/search.php, version 06/2021 (2021).
71. Welton, B. J. *Cetorhinus* cf. *C. maximus* (Gunnerus)(Lamniformes: Cetorhinidae), a basking shark from the Late Miocene empire formation, Coos Bay, Oregon. *Bull. - South. Calif. Acad. Sci.*, **112,** 74-92 (2013).
72. Trystram, C., Rogers, K. M., Soria, M. & Jaquemet, S. Feeding patterns of two sympatric shark predators in coastal ecosystems of an oceanic island. *Can. J. Fish. Aquat. Sci.* **74,** 216-227 (2017).
73. Weigmann, S. Annotated checklist of the living sharks, batoids and chimaeras (Chondrichthyes) of the world, with a focus on biogeographical diversity. *J. Fish Biol.*, ***88*****,** 837-1037 (2016).
74. Cliff, G. & Dudley, S. F. J. Sharks caught in the protective gill nets off Natal, South Africa. 4. The bull shark *Carcharhinus leucas* (Valenciennes). *Afr. J. Mar. Sci.* **10,** 253-270 (1991).
75. Smale, M. J. Occurrence and feeding of three shark species, *Carcharhinus brachyurus*, *C. obscurus* and *Sphyrna zygaena*, on the Eastern Cape coast of South Africa. *Afr. J. Mar. Sci.* **11,** 31-42 (1991).
76. Hoffmayer, E. R. & Parsons, G. R. Food habits of three shark species from the Mississippi Sound in the northern Gulf of Mexico. *Southeast. Nat.* **2,** 271-280 (2003).
77. McElroy, W. D. et al. Food habits and ontogenetic changes in the diet of the sandbar shark (*Carcharhinus plumbeus*) in Hawaii. *Environ. Biol. Fishes* **76,** 81-92 (2006).
78. France, R., Loret, J., Mathews, R. & Springer, J. Longitudinal variation in zooplankton $\delta^{13}C$ through the Northwest Passage: inference for incorporation of sea-ice POM into pelagic foodwebs. *Polar Biol.* **20,** 335-341 (1998).
79. Dorado, S., Rooker, J. R., Wissel, B. & Quigg, A. Isotope baseline shifts in pelagic food webs of the Gulf of Mexico. *Mar. Ecol. Prog. Ser.* **464,** 37-49 (2012).

80. Abrantes, K. G., Barnett, A., Baker, R. & Sheaves, M. Habitat-specific food webs and trophic interactions supporting coastal-dependent fishery species: an Australian case study. *Rev. Fish Biol. Fish.* **25,** 337-363 (2015).
81. De Lecea, A. M., Fennessy, S. T. & Smit, A. J. Processes controlling the benthic food web of a mesotrophic bight (KwaZulu-Natal, South Africa) revealed by stable isotope analysis. *Mar. Ecol. Prog. Ser.* **484,** 97-114 (2013).
82. De Lecea, A. M., Cooper, R. & Smit, A. J. Identifying the drivers of the pelagic ecosystem of an oligotrophic bight (KwaZulu–Natal, South Africa) using stable isotopes ($\delta^{13}C$, $\delta^{15}N$) and C:N ratio analysis. *Mar. Freshw. Res.* **67,** 1750-1761 (2015).
83. Le Croizier, G., et al. Mercury isotopes as tracers of ecology and metabolism in two sympatric shark species. *Environ. Pollut.* **265,** 114931 (2020). https://doi.org/10.1016/j.envpol.2020.114931
84. Kocsis, L., Vennemann, T. W., Ulianov, A. & Brunnschweiler, J. M. Characterizing the bull shark *Carcharhinus leucas* habitat in Fiji by the chemical and isotopic compositions of their teeth. *Environ. Biol. Fishes* **98,** 1609-1622 (2015).
85. Müller, W., et al. Enamel mineralization and compositional time-resolution in human teeth evaluated via histologically-defined LA-ICPMS profiles. *Geochim. Cosmochim. Acta* **255,** 105-126. (2019).
86. Bury, N. R., Walker, P. A. & Glover, C. N. Nutritive metal uptake in teleost fish. *J. Exp. Biol.* **206,** 11-23 (2003).
87. Kocsis, L., Trueman, C. N. & Palmer, M. R. Protracted diagenetic alteration of REE contents in fossil bioapatites: direct evidence from Lu–Hf isotope systematics. *Geochim. Cosmochim. Acta* **74,** 6077-6092 (2010).
88. McCormack, J. M., Bahr, A., Gerdes, A., Tütken, T. & Prinz-Grimm, P. Preservation of successive diagenetic stages in Middle Triassic bonebeds: Evidence from in situ trace element and strontium isotope analysis of vertebrate fossils. *Chem. Geol*. **410,** 108-123 (2015).
89. Sharp, Z. *Principles of Stable Isotope Geochemistry*. (Open Educational Resources, Albuquerque, 2017).
90. Pimiento, C., Cantalapiedra, J. L., Shimada, K., Field, D. J. & Smaers, J. B. Evolutionary pathways toward gigantism in sharks and rays. *Evolution* **73,** 588-599 (2019).
