# Supplementary material for: Trophic position of Otodus megalodon and great white sharks through time revealed by zinc isotopes
Source: Nat Commun. 2022 May 31;13:2980. doi: 10.1038/s41467-022-30528-9 (PMC9156768; doi:10.1038/s41467-022-30528-9)
Supplement: Supplementary file 3 — Description of Additional Supplementary Files [file 41467_2022_30528_MOESM3_ESM.docx]

**Supplementary Data 1:** Background information and geochemical data for each extant and fossil tooth analysed. Information includes taxonomic information, δ^66^Zn, δ^13^C_coll_, δ^15^N_coll_ and δ^18^O_P_ values, mean FishBase (ref.^17^) trophic level for extant species, total body length estimates of individuals of fossil *Otodus chubutensis*, *O. megalodon*, *Carcharodon hastalis*, and *C. carcharias* based on tooth crown height, museum catalogue numbers, locality, age, and stratigraphic context. For extant samples the same museum catalogue number indicates the same individual, except for aquaria teeth which likely belong to multiple individuals in the tank and JGU GW OS2021/19 and JGU GW OS2021/20 (different teeth of the same individual). Samples are deposited at the Calvert Marine Museum, Solomons, Maryland, USA (CMM); Osteological Collection, Institute of Geosciences, Johannes Gutenberg-University, Mainz, Germany (JGU GW); Natural History Museum of Los Angeles County, California, USA (LACM); Massachusetts Natural History Collections, University of Massachusetts, Amherst, Massachusetts, USA (MNHC); Zoologisk Museum, Universitetet i Bergen, Bergen, Norway (ZMUB); and the Field Museum of Natural History, Chicago, Illinois, USA (FMNH).
