# Supplementary material for: Trophic position of Otodus megalodon and great white sharks through time revealed by zinc isotopes
Source: Nat Commun. 2022 May 31;13:2980. doi: 10.1038/s41467-022-30528-9 (PMC9156768; doi:10.1038/s41467-022-30528-9)
Supplement: Supplementary file 5 — Reporting Summary [file 41467_2022_30528_MOESM5_ESM.pdf]

nature portfolio

Corresponding author(s): Jeremy McCormack

Last updated by author(s): Apr 21, 2022

Reporting Summary

Nature Portfolio wishes to improve the reproducibility of the work that we publish. This form provides structure for consistency and transparency in reporting. For further information on Nature Portfolio policies, see our Editorial Policies and the Editorial Policy Checklist.

Statistics

For all statistical analyses, confirm that the following items are present in the figure legend, table legend, main text, or Methods section.

n/a	Confirmed	
☐	☒	The exact sample size (*n*) for each experimental group/condition, given as a discrete number and unit of measurement
☐	☒	A statement on whether measurements were taken from distinct samples or whether the same sample was measured repeatedly
☐	☒	The statistical test(s) used AND whether they are one- or two-sided<br>*Only common tests should be described solely by name; describe more complex techniques in the Methods section.*
☐	☒	A description of all covariates tested
☐	☒	A description of any assumptions or corrections, such as tests of normality and adjustment for multiple comparisons
☐	☒	A full description of the statistical parameters including central tendency (e.g. means) or other basic estimates (e.g. regression coefficient) AND variation (e.g. standard deviation) or associated estimates of uncertainty (e.g. confidence intervals)
☐	☒	For null hypothesis testing, the test statistic (e.g. *F*, *t*, *r*) with confidence intervals, effect sizes, degrees of freedom and *P* value noted<br>*Give P values as exact values whenever suitable.*
☒	☐	For Bayesian analysis, information on the choice of priors and Markov chain Monte Carlo settings
☒	☐	For hierarchical and complex designs, identification of the appropriate level for tests and full reporting of outcomes
☒	☐	Estimates of effect sizes (e.g. Cohen's *d*, Pearson's *r*), indicating how they were calculated

Our web collection on statistics for biologists contains articles on many of the points above.

Software and code

Policy information about availability of computer code

Data collection	Thermo Scientific Mass Spectrometry Software.
Data analysis	All statistical analyses were conducted using the free program R software (R version 4.0.2)

For manuscripts utilizing custom algorithms or software that are central to the research but not yet described in published literature, software must be made available to editors and reviewers. We strongly encourage code deposition in a community repository (e.g. GitHub). See the Nature Portfolio guidelines for submitting code & software for further information.

Data

Policy information about availability of data

All manuscripts must include a data availability statement. This statement should provide the following information, where applicable:

- Accession codes, unique identifiers, or web links for publicly available datasets
- A description of any restrictions on data availability
- For clinical datasets or third party data, please ensure that the statement adheres to our policy

All data generated during this study are included in this published article (and its supplementary information files).

Field-specific reporting

Ecological, evolutionary & environmental sciences study design

All studies must disclose on these points even when the disclosure is negative.

Study description	We studied zinc isotopes in extant and fossil elasmobranch and teleost teeth as a dietary proxy. We demonstrate the use of zinc isotopes as a trophic level proxy for extant fish and show that the pristine dietary zinc isotope signal is preserved in fossil tooth enameloid over millions of years. We then interpret the zinc isotope values of extinct shark species with a special emphasis on reconstructing the ecology of extinct megatooth sharks.
Research sample	We studied extant and fossil teeth of various elasmobranch and teleost species. Extant samples come from a variety of geographic locations including aquarium samples. Fossil samples come from Germany, Malta, Japan, North Carolina (USA) and Florida (USA), covering the Early Miocene, Miocene-Pliocene transition, and the Early Pliocene. Extant samples were chosen to cover a high range of trophic levels, taxa, and geographic locations. Fossil samples were chosen to cover a high range of temporal and taxonomic diversity. Every fossil specimen comes from a non-embryonic individual and falls in the size range that would represent medium (non-neonate young) to large (old) individuals. A full description of taxonomy, museum catalogue numbers, locality, age, and stratigraphic context can be found in Supplementary Data 1.
Sampling strategy	Samples were chosen to include multiple taxa and achieve a high geographic and age spacing. Enameloid samples were abraded from the top surface using a dental drill for subsequent zinc and oxygen isotope analyses. Powdered dentine samples, for collagen carbon and nitrogen isotope analysis, were collected from all modern teeth and bones using a low-speed handheld drill with a diamond-tipped bit.
Data collection	Mass spectrometry: Jeremy McCormack, Sora L. Kim, Molly Karnes, Sarah Pederzani. Electron microprobe analysis: Niels Jöns
Timing and spatial scale	The analyses are not time dependent.
Data exclusions	All data are reported.
Reproducibility	All isotopic measurements included the analyses of internationally recognised standards and sample replicate analyses. All extant and fossil samples are formally cataloged and curated at proper repository institutions under public trust.
Randomization	Does not apply to this type of study.
Blinding	Does not apply to this type of study.

Did the study involve field work? ☐ Yes ☒ No

Reporting for specific materials, systems and methods

We require information from authors about some types of materials, experimental systems and methods used in many studies. Here, indicate whether each material, system or method listed is relevant to your study. If you are not sure if a list item applies to your research, read the appropriate section before selecting a response.

Materials & experimental systems

n/a	Involved in the study
☒	☐ Antibodies
☒	☐ Eukaryotic cell lines
☐	☒ Palaeontology and archaeology
☒	☐ Animals and other organisms
☒	☐ Human research participants
☒	☐ Clinical data
☒	☐ Dual use research of concern

Methods

n/a	Involved in the study
☒	☐ ChIP-seq
☒	☐ Flow cytometry
☒	☐ MRI-based neuroimaging

Palaeontology and Archaeology

Specimen provenance	Fossil material comes from the Baltringer Formation of Germany (Early Miocene), Globigerina Limestone of Malta (Early Miocene), Na-arai Formation of Japan (Early Pliocene), Pungo River and Yorktown formations North Carolina (USA, Early Miocene and Early Pliocene) and Peace River and Tamiami formations of Florida (USA, Miocene-Pliocene transition). Additional provenance information for the samples is provided in the supplementary material. Permissions to conduct isotopic analyses were provided by the respective repository institutions the specimen are curated in through loan agreements.
Specimen deposition	All extant and fossil material analysed in this study is deposited in the following repository institutions: Samples are deposited at the Calvert Marine Museum, Solomons, Maryland, USA (CMM); Osteological Collection, Institute of Geosciences, Johannes Gutenberg-University, Mainz, Germany (JGU GW); Natural History Museum of Los Angeles County, California, USA (LACM); Massachusetts Natural History Collections, University of Massachusetts, Amherst, Massachusetts, USA (MNHC); Zoologisk Museum, Universitetet i Bergen, Bergen, Norway (ZMUB); and the Field Museum of Natural History, Chicago, Illinois, USA (FMNH). See also Supplementary Data 1.
Dating methods	No new dates were obtained as part of this research. References for previously published stratigraphic context and dates are provided in the supplementary material.

☒ Tick this box to confirm that the raw and calibrated dates are available in the paper or in Supplementary Information.

Ethics oversight	Ethical approval was not required for this study. All analyses were conducted on samples that are formally cataloged and curated at proper repository institutions under public trust.

Note that full information on the approval of the study protocol must also be provided in the manuscript.
